# Phenolic Composition and Antioxidant Activity of Purple Sweet Potato (*Ipomoea batatas* (L.) Lam.): Varietal Comparisons and Physical Distribution

**DOI:** 10.3390/antiox10030462

**Published:** 2021-03-16

**Authors:** Yeong Ran Im, Inhwan Kim, Jihyun Lee

**Affiliations:** Department of Food Science and Technology, Chung-Ang University, Anseong 17546, Korea; dladudfks53@naver.com (Y.R.I.); hgodos@hanmail.net (I.K.)

**Keywords:** purple sweet potato, outer layer and inner layer, anthocyanins, polyphenols, UHPLC-(ESI)-qTOF, UHPLC-(ESI)-QqQ, antioxidant activity

## Abstract

The outer layer of purple sweet potato is removed during processing; however, this layer serves as a potential source of phenolics, especially anthocyanins. Herein, the phenolic composition and antioxidant activity were determined for the inner and outer layers of five purple sweet potato cultivars (‘Sinjami’, ‘Jami’, ‘Danjami’, ‘Yeonjami’, and ‘Borami’) harvested in Korea. Anthocyanins were identified using ultra-high-performance liquid chromatography coupled with electrospray ionization quadrupole time-of-flight mass spectrometer (UHPLC-(ESI)-qTOF-MS) and ultra-high-performance liquid chromatography-linear ion trap mass spectrometer (UHPLC-Ion trap-MS), and their composition was quantified using HPLC-coupled with diode array detector (DAD). Non-anthocyanin phenolic compounds (phenolic acids and flavonols) were quantified using UHPLC-(ESI)-triple quadrupole (QqQ). A total of 20 anthocyanins, including non-acylated or acylated peonidin, cyanidin, and pelargonidin glycosides, were identified. Peonidin 3-caffeoyl-*p*-hydroxybenzoyl sophoroside-5-glucoside was the major anthocyanin, with the highest level in the ‘Sinjami’ cultivar (outer; 12,366 mg/kg DW, inner; 14,832 mg/kg DW). Additionally, 12 phenolic acids and 6 flavonols (quercetin derivatives) were identified, with the outer layers of all cultivars displaying higher total levels than the inner layers. ‘Sinjami’ and ‘Jami’ had higher phenolic acid and quercetin derivative content and antioxidant activities than the other three cultivars (*p* < 0.05). Thus, the outer layers of ‘Sinjami’ and ‘Jami’ cultivars could be potential sources of anthocyanins and other phenolics.

## 1. Introduction

Anthocyanins are natural pigments responsible for the blue, red, or purple color in fruits and vegetables [1,2]. Furthermore, anthocyanins have protective effects in conditions such as cancer, inflammation, and cardiovascular diseases [3]. Safety concerns, such as adverse behavioral and neurological effects, of synthetic colorants commonly used in the food industry, have been raised over the past few years. In response to increasing consumer and food manufacturer demand for natural colorants, anthocyanins, having possible health benefits, could be promising alternatives to synthetic colorants as they have been consumed for centuries without any adverse effects [4,5].

Sweet potato (*Ipomoea batatas* L.) belongs to the family Convolvulaceae. It is believed to have originated from Latin America. In the 16th century, sweet potato was introduced to Asia, Africa, and Europe. Worldwide sweet potato production is estimated to be 105 million tons, with Asia accounting for 74.7% (79 million tons) of global production in 2016 [6,7]. Purple sweet potato (*Ipomoea batatas* (L.) Lam.) is a rich source of anthocyanins (515–1747 mg/kg fresh weight (FW)) [8]. The major anthocyanins in purple sweet potato are peonidin 3-sophoroside-5-glucoside and cyanidin 3-sophoroside-5-glucoside, which are mono- or di-acylated with caffeic, ferulic, and *p*-hydroxybenzoic acids [9]. Acylated anthocyanins account for more than 98% of total anthocyanin contents in purple sweet potato [10,11]. Although anthocyanins are natural colorants preferred by consumers over synthetic colorants, they are not stable during food processing. Previous studies showed that the stability of acylated anthocyanins during processing increases with an increasing number of acyl groups [12,13]. 

In vitro and in vivo studies have shown that purple sweet potato anthocyanins have antioxidant, anticancer, and antimutagenicity activities [14,15,16]. In addition to anthocyanin, purple sweet potato also contains chlorogenic, dicaffeoylquinic, caffeic, and ferulic acids [17,18]. Phenolic compounds, such as ferulic and caffeic acids, increase anthocyanin stability via intermolecular copigmentation by shielding the flavylium cation from nucleophilic attack by water [19,20].

Anthocyanin composition is dependent on the food source and cultivar used. Varietal differences of anthocyanins were previously reported for ‘Borami’, ‘Jami’, ‘Sinjami’, and ‘Yeonjami’ purple sweet potato cultivars [21,22]; however, anthocyanin composition of other commercially important purple sweet potato cultivars in Korea, including ‘Danjami’, is unknown. There is also limited information on other phenolic compounds in purple sweet potato. More importantly, the physical distribution of phenolic compounds in purple sweet potato is unknown. Most studies have used a whole purple sweet potato to determine anthocyanins and other phenolic compounds [23,24]. When a purple sweet potato is cut horizontally, a white round band divides the outer and inner layers; the outer layer is often discarded during processing [25]. However, the outer layer is a possible commercial source of phenolics, especially anthocyanin pigments. 

To address these issues, varietal differences and physical distribution (inner and outer layers) of anthocyanins and non-anthocyanin phenolic compounds were investigated in five commercially important purple sweet potato cultivars; their antioxidant activity was also determined.

## 2. Materials and Methods

### 2.1. Chemicals and Reagents

Anthocyanin standards (cyanidin-3-*O*-glucoside, peonidin-3-*O*-glucoside, and pelargonidin-3-*O*-glucoside) were purchased from Extrasynthese (Lyon Nord, France). Gallic and formic acid, Trolox, and 2,2-diphenyl-1-picrylhydrazyl (DPPH) were obtained from Sigma–Aldrich (S4t. Louis, MO, USA). HPLC-grade methanol and acetonitrile were purchased from Burdick & Jackson (Muskegon, MI, USA). The water used in this study was obtained from a water purification system (Milli-Q Direct 8, Merck Millipore, Billerica, MA, USA).

### 2.2. Purple Sweet Potato Samples

**Figure 1:**
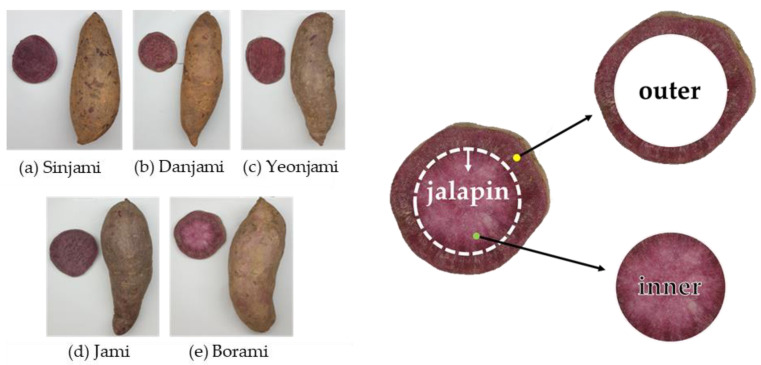
Images of the five purple sweet potato cultivars harvested in Korea and a diagram indicating the jalapin, outer, and inner layers of Borami, defined in this study.

Five purple sweet potato cultivars (‘Sinjami’, ‘Jami’, ‘Yeonjami’, ‘Danjami’, and ‘Borami’) were obtained from a research orchard of the Bioenergy Crop Research Center of the National Institute of Crop Science (Muan-gun, Korea; 34°58′09.3″ N 126°27′18.3″ E) in Korea in November 2019. Each cultivar weighed 5 kg, and 20–25 tubers were used for analysis. Purple sweet potato images are shown in Figure 1. All samples used were peeled and cut into 1.5 cm slices. Inner and outer layers were divided by the jalapin layer of the cross-section. Samples were freeze-dried and stored at −80 °C until analysis. Before analysis, freeze-dried samples were ground into a fine powder using a high-speed blender (Nutri Ninja Duo Auto-iQ, SharkNinja, Shenzhen, China), and the powder was passed through a 0.85-mm sieve to obtain uniform size.

### 2.3. Extrtaction of Anthocyanin and Non-Anthocyanin Phenolic Compounds

Five milliliters of 0.2% HCl in methanol was added to 500 mg lyophilized purple sweet potato powder. The mixture was sonicated for 15 min using a sonicator (CPX3800, Branson Ultrasonic Corporation, Danbury, CT, USA) and centrifuged at 11,124× *g* for 20 min at 4 °C. This procedure was repeated four times until the purple color of the supernatant disappeared. All supernatant extracts were transferred into a volumetric flask and made up to 30 mL with extraction solvents. An aliquot (10 mL) of the extract was evaporated under nitrogen using a nitrogen concentrator (MG-2200, EYELA, Tokyo, Japan) and re-dissolved in aqueous 0.2% HCl (1.5 mL). Solid-phase extraction (SPE) was performed for further anthocyanin purification, as described previously with slight modifications [26]. After SPE, cartridges were preconditioned with 6 mL methanol and 6 mL aqueous 0.2% HCl, and the extract was loaded onto 1 g (6 cc) Sep-Pak C18 cartridges (Waters, Milford, MA, USA). After loading, the cartridge was washed with 6 mL aqueous 0.2% HCl. Anthocyanins were eluted with 8 mL 0.2% HCl in methanol. The eluted anthocyanin fraction was concentrated to dryness using the nitrogen concentrator (MG-2200), re-dissolved in 1 mL water and methanol (1:1, *v*/*v*), and filtered using a 0.22-μm polyvinylidene fluoride (PVDF) filter before HPLC and LC-MS analyses.

For the analysis of non-anthocyanin phenolic compounds, 500 mg of lyophilized purple sweet potato powder was mixed with 10 mL of 80% aqueous methanol solution, as described previously [27]. The mixture was sonicated for 15 min using the sonicator (CPX3800) and centrifuged at 11,124× *g* for 15 min at 4 °C. Supernatants were concentrated using a Speedvac concentrator (SPD 2010, Thermo Scientific, Waltham, MA, USA) and re-dissolved in 1 mL aqueous 0.1% formic acid. The re-dissolved concentrates were filtered through a 0.22-μm PVDF filter. Anthocyanin and non-anthocyanin analyses were performed within 1–2 days after extraction. All extraction procedures were performed in triplicate (n = 3).

### 2.4. Identification of Anthocyanins Using a UHPLC-(ESI)-qTOF and UPLC-Iontrap

Anthocyanins were identified using an Acquity ultra-high-performance liquid chromatography (UHPLC) system coupled to a Synapt G2-Si HDMS accurate mass quadrupole time-of-flight mass spectrometer (qTOF MS) in positive electrospray ionization (ESI) mode (Waters, Milford, MA, USA). Anthocyanin compounds were separated on a Waters Acquity BEH C18 column (2.1 mm × 100 mm, 1.7 μm). Mobile phases were 0.1% formic acid in water (A) and 0.1% formic acid in acetonitrile (B). The gradient program was as follows: 0–8 min, 5–40% (B); 9.0–9.2 min, 80–100% (B); and 10.2–10.7 min, 30–70% (B). The column temperature was maintained at 30 °C. The injection volume was 5 μL, and the flow rate was 0.4 mL/min. The MS1 mode of qTOF was used for anthocyanin analysis. Cone and capillary voltages were set to 30 kV and 15 kV, respectively. Source and desolvation temperatures were 120 °C and 500 °C, respectively. The mass scan range was 60–1400 *m*/*z*.

For anthocyanin identification, an in-house accurate mass database of all potential anthocyanins was built, and the database included previously reported anthocyanins in purple sweet potato cultivars and possible anthocyanins present in samples. The theoretical mass of each possible anthocyanin was calculated based on the molecular formula. Potential anthocyanins in extracts were identified based on a comparison of measured accurate masses to the calculated theoretical masses, using Waters MassLynx MS software version 4.1. Identification was assisted by MS^n^ data-dependent mode of LTQ Velos Pro (ThermoFisher Scientific, Austin, TX, USA) linear ion trap mass spectrometer (UHPLC-Ion trap-MS) to determine fragments of the identified anthocyanins. For UHPLC-Ion trap-MS, mobile phases were 0.5% formic acid in water (A) and 0.5% formic acid in acetonitrile (B). Anthocyanins were separated on a Zorbax Eclipse XDB-C18 column (4.6 mm × 250 mm, 5 µm; Agilent Technologies, Santa Clara, CA, USA). The mobile phase gradient program used was as follows: 0–6.5 min, 10–12% (B); 6.5–10.5 min, 12–13% (B); 10.5–33 min, 13–17% (B); 33–60 min, 17%–65% (B); and 60–70 min, 65–95% (B). The injection volume was 20 µL, and the column oven temperature was maintained at 30 °C. The flow rate was 1.0 mL/min; half of the flow was diverted to the MS, and the rest was moved to waste. Capillary and source heater temperatures were 275 °C and 250 °C, respectively. Source voltage was 5.0 kV, and the S-lens RF level was 60.00%. Pseudomolecular ions were scanned in positive mode full scan and MS^n^ data-dependent mode of UHPLC-Ion trap-MS with a mass range of 50–2000 *m*/*z*.

### 2.5. Quantification of Anthocyanins Using HPLC-DAD

Anthocyanin content was quantified using an Agilent 1260 Infinity II HPLC system (Agilent Technologies) with a diode array detector (DAD) [28]. Anthocyanin compounds were separated on a Zorbax Eclipse XDB-C_18_ column (4.6 × 250 mm, 5 µm; Agilent Technologies). Mobile phases were 1.5% formic acid in water (A) and 1.5% formic acid in acetonitrile (B). The same gradient program as that for the UHPLC-Ion trap-MS method was used for quantifying anthocyanins using HPLC-DAD. The injection volume was 20 µL, and the column oven temperature was maintained at 30 °C. The flow rate was 1.0 mL/min, and detection was conducted at 520 nm.

### 2.6. Determination of Non-Anthocyanin Phenolic Composition Using UHPLC-(ESI)-QqQ

The non-anthocyanin phenolic compounds were determined using an Agilent 1290 Infinity II UHPLC coupled with an Agilent 6470 triple quadrupole (QqQ) mass spectrometer (Agilent Technologies) in negative ESI mode. Phenolic compounds were separated at 30 °C on a Poreshell 120 EC-C18 column (2.1 mm × 100 mm, 2.7 µm; Agilent Technologies). Mobile phases were 0.1% formic acid in water (A) and 0.1% formic acid in acetonitrile (B). The mobile phase gradient program used was as follows: 0–5 min, 5–10% (B); 5–8 min, 10–12% (B); 8–10 min, 12–15% (B); 10–15 min, 15% (B); 15–18 min, 15–55% (B); 18–20 min, and 55–90% (B). The flow rate was 0.4 mL/min, and the injection volume was 5 μL. Full scan mode with a range of 100 to 1000 *m*/*z* was applied to identify all possible phenolic compounds in negative mode. A gas temperature of 150 °C, capillary voltage of 4000 V, drying gas flow of 8 L/min, nebulizer pressure of 30 psi, nozzle voltage of 500 V, sheath gas temperature of 300 °C, and sheath gas flow of 10 L/min were used.

The multiple reaction monitoring (MRM) mode was used to determine the phenolic compounds. The fragment voltage (FV) and collision energy (CE) were optimized for each phenolic compound to detect product ions at maximum intensity. MRM transitions from the precursor ion to the product ion were selected as follows: caffeic acid, *m*/*z* 179 > *m*/*z* 135 (FV:100 V and CE: 15 V); *trans*-ferulic acid and *cis*-ferulic acid, *m*/*z* 193 > *m*/*z* 134 (FV: 100 V, CE: 15 V); chlorogenic acid and caffeoylquinic acids, *m*/*z* 353 > *m*/*z* 191 (FV: 180 V, CE: 15 V); dicaffeoylquinic acids, *m*/*z* 515 > *m*/*z* 191 (FV: 180 V, CE: 30 V); p-coumaric acid, *m*/*z* 163 > *m*/*z* 119 (FV: 100 V, CE: 15 V); quercetin 3-*O*-galactoside and quercetin 3-*O*-glucoside, *m*/*z* 463 > *m*/*z* 301; and quercetin diglucosides, *m*/*z* 625 > 301 (FV: 180 V, CE: 30 V). Absolute quantification of caffeic acid, ferulic acid, chlorogenic acid, p-coumaric acid, quercetin 3-*O*-galactoside, and quercetin-3-*O*-glucoside was achieved using authentic standards. Quercetin diglucosides and *cis*-ferulic acid were quantified using standard curves of quercetin 3-*O*-glucoside and *trans*-ferulic acid, respectively. Caffeoylquinic acid and dicaffeoylquinic acids were quantified using a chlorogenic acid standard curve. Quantification results are expressed as mg/kg dry weight (DW).

### 2.7. Total Phenolic Content Measurement

Total phenolic content (TPC) was measured using the Folin–Ciocalteu reagent, as described previously [28]. Diluted sample extracts (40 µL) were added to 50 µL of 1 N Folin–Ciocalteu reagent and allowed to react for 1 min. Approximately 160 µL of 2% sodium carbonate (Na_2_CO_3_) solution was added to the mixture. After 30 min of incubation at 25 °C, absorbance at 700 nm was measured using a spectrophotometer (Multiskan GO, Thermo Scientific, Waltham, MA, USA). TPC was expressed as mg gallic acid equivalent/100 g dried weight (mg GAE/100 g DW).

### 2.8. Antioxidant Activities

Antioxidant activities of purple sweet potato extracts were determined by measuring 2,2-diphenyl-1-picrylhydrazyl (DPPH) radical scavenging activity and performing 2,2′-azino-bis (3-ethylbenzothiazoline 6-sulphonic acid) (ABTS) assay. DPPH radical scavenging activity was determined as described previously [26,29]. Sample extracts (150 µL) were reacted with 200 µL of 0.15 mM DPPH in ethanol at 25 °C for 15 min. The absorbance was measured at 517 nm.

An ABTS assay was conducted as described previously, with some modifications [30]. ABTS radical cation (ABTS^•+^) solution was produced by reacting a 7 mM ABTS stock solution with 2.45 mM potassium persulfate (1:1, *v*/*v*) for 16 h before use, and the solution was diluted with methanol to obtain a starting absorbance of 0.77 at 734 nm. Approximately 300 μL ABTS^•+^ solution was mixed with 20 μL of sample extracts, and the absorbance was measured at 734 nm after 10 min. Results of the DPPH and ABTS assays were expressed as mg Trolox equivalent antioxidant capacity (TEAC)/g DW.

### 2.9. Statistical Analysis

Statistical analyses, including one-way analysis of variance (ANOVA), followed by Duncan’s post hoc test and Pearson’s correlation analysis, were performed using IBM SPSS Statistics 25 (v. 25.0, SPSS, Inc., Chicago, IL, USA). Principal component analysis (PCA) and heatmap of anthocyanins and non-anthocyanins, determined by HPLC-DAD and UHPLC-QqQ, were performed using XLSTAT (Microsoft Excel Add-in Software, New York, NY, USA).

## 3. Results

### 3.1. Identification of Anthocyanins in Purple Sweet Potato Extracts Using UHPLC-(ESI)-qTOF and UHPLC-Iontrap

Twenty anthocyanin compounds were identified (Table 1). Figure S1 shows representative HPLC-DAD chromatograms of anthocyanin extracts from the inner and outer layers of Yeonjami and Jami. As authentic standards are currently not commercially available for all identified anthocyanins, anthocyanins were tentatively identified based on their accurate mass and fragments analyzed using UHPLC-qTOF and UHPLC-Ion Trap-MS, respectively, and by the elution order of anthocyanin peaks, as reported previously [21,22,31]. The mass error was below 6.3 ppm.

Most anthocyanins are conjugated forms of cyanidin, peonidin, and pelargonidin. The identified anthocyanins included cyanidin 3-sophoroside-5-glucoside, peonidin 3-sophoroside-5-glucoside, and pelargonidin 3-sophoroside-5-glucoside acylated with phenolic acids, including caffeic acid (*m*/*z* 162), ferulic acid (*m*/*z* 176), and *p*-hydroxybenzoic acid (*m*/*z* 120), as reported previously [21,22]. The anthocyanin composition was specific to purple sweet potato cultivars. For example, pelargonidin-based anthocyanins (peaks 2, 9, 11, and 19) were only found in the Borami cultivar.

Anthocyanins produced their characteristic fragment ion *m*/*z* of each anthocyanin aglycone after the loss of their conjugates. Cyanidin-related anthocyanins (peaks 1, 4, 5, 8, 12, 13, and 15) produced a fragment ion at *m*/*z* 287, corresponding to cyanidin aglycone after the loss of the conjugate [M conjugate]^+^. Cyanidin 3-sophoroside-5-glucoside, pelargonidin 3-sophoroside-5-glucoside, and peonidin 3-sophoroside-5-glucoside (peaks 1, 2, and 3) produced characteristic fragment ions of their aglycones after losing one or more glucose moieties (loss of *m*/*z* 162 per glucose moiety). Fragment ions at *m*/*z* 271 and *m*/*z* 301 indicated pelargonidin aglycone and peonidin aglycone, respectively. Cyanidin 3-hydroxybenzoyl sophoroside-5-glucoside and peonidin 3-hydroxybenzoyl sophoroside-5-glucoside (peaks 4 and 6) produced fragment ions after the loss of *m*/*z* 162 and *m*/*z* 444, corresponding to glucose and *p*-hydroxybenzoyl sophroside, respectively.

Anthocyanins containing caffeoyl sophoroside at the C3 position of aglycone (peaks 5, 7, 9, 12, and 14) produced characteristic fragment ions, resulting from the loss of one glucose moiety (*m*/*z* 162) at the C5 position and caffeoyl sophorose moiety (*m*/*z* 486). Anthocyanins containing feruloyl sophoroside at the C3 position of aglycone (peaks 8, 10, and 11) produced characteristic fragment ions, resulting from the loss of one glucose moiety (*m*/*z* 162) at the C5 position and feruloyl sophorose moiety (*m*/*z* 500). Anthocyanins containing caffeoyl-*p*-hydroxybenzoyl sophoroside at the C3 position of aglycone (peaks 13 and 17) produced characteristic fragment ions, resulting from losing one glucose (*m*/*z* 162) at the C5 position and caffeoyl-*p*-hydroxybenzoyl sophorose (*m*/*z* 606). Anthocyanins containing caffeoyl–feruloyl sophoroside at the C3 position of aglycone (peaks 15, 18, and 19) produced characteristic fragment ions, resulting from losing one glucose moiety (*m*/*z* 162) at the C5 position and c caffeoyl–feruloyl sophorose moiety (*m*/*z* 662). Peonidin 3-dicaffeoyl sophoroside-5-glucoside (peak 16) produced a characteristic fragment ion at *m*/*z* 949 after losing *m*/*z* 162 corresponding to one glucose moiety and at *m*/*z* 463 after losing *m*/*z* 648 corresponding to dicaffeoyl sophorose at the C5 position of aglycone. Peonidin 3-feruloyl *p*-hydroxybenzoyl sophoroside-5-glucoside (peak 20) produced a fragment ion at *m*/*z* 921 after losing *m*/*z* 162 corresponding to one glucose moiety and at *m*/*z* 463 after losing *m*/*z* 620 corresponding to feruloyl-*p*-hydroxybenzoyl sophorose.

### 3.2. Quantification of Anthocyanins in Purple Sweet Potato Cultivars

Table 2 shows anthocyanin content in the outer and inner layers of the five cultivars. Individual anthocyanin content was expressed as mg cyanidin-3-*O*-glucoside equivalent eq./kg DW, mg peonidin-3-*O*-glucoside eq./kg DW, and mg pelargonidin-3-*O*-glucoside eq./kg DW based on their anthocyanidin structures. F- and *p*-values of all purple sweet potato samples for individual phenolic contents, total phenolic content (TPC), and antioxidant activity by multivariate ANOVA are shown in Table S1. Total anthocyanin content (sum of individual anthocyanin contents) ranged from 7477 to 34,569 mg/kg DW and varied among cultivars. For the outer layer, ‘Sinjami’ cultivar showed the highest total anthocyanin content (30,446 mg/kg DW), followed by ‘Jami’ (20,365 mg/kg DW), ‘Yeonjami’ (14,010 mg/kg DW), ‘Borami’ (13,732 mg/kg DW), and ‘Danjami’ (8421 mg/kg DW). ‘Sinjami’ cultivar also had the highest total anthocyanin content in the inner layer (34,569 mg/kg DW), followed by Jami (19,352 mg/kg DW), ‘Yeonjami’ (12,020 mg/kg DW), ‘Borami’ (8833 mg/kg DW), and ‘Danjami’ (7477 mg/kg DW). Notably, the outer layer, which is typically removed during purple sweet potato processing, had higher total anthocyanin contents than the inner layer of all cultivars except the ‘Sinjami’ cultivar.

Notable differences in mono-acylated anthocyanin and di-acylated anthocyanin content were found, depending on the cultivar. ‘Sinjami’, ‘Danjami’, and ‘Yeonjami’ contained 72–77% di-acylated anthocyanin contents, whereas ‘Jami’ and ‘Borami’ contained higher (90–95%) di-acylated anthocyanin contents, regardless of tissue layers (i.e., inner and outer layers). However, ‘Sinjami’, ‘Danjami’, and ‘Yeonjami’ contained a higher mono-acylated anthocyanin ratio (21–24%) than the other two cultivars (4–7%). The proportion of non-acylated anthocyanins (peaks 1, 2, and 3) was less than 6% of the total anthocyanin content in all tested cultivars. The non-acylated and acylated anthocyanin ratios for Korean purple sweet potato cultivars in our study were similar to those reported by a previous study, in which di-acylated, mono-acylated, and non-acylated anthocyanins contributed 81.5–90.2%, 7.6–11.5%, and 1.8–7% to the total anthocyanin content in Chinese purple sweet potato, respectively [32]. Predominant anthocyanin species, i.e., di-acylated and mono-acylated anthocyanins, ranged from 52.1% to 71.0% and from 27.2% to 41.3%, respectively, whereas non-acylated anthocyanins accounted for less than 6.4% of the total anthocyanin contents in four Korean purple sweet potato varieties (‘Sinjami’, ‘Jami’, ‘Borami’, and ‘Mokpo 62’) [22]. A previous study showed that anthocyanins di-acylated with phenolic acids are more stable to heat, light, and H_2_O_2_ than mono-acylated anthocyanins [33].

The total content of peonidin-based anthocyanins ranged from 6544 to 26,483 mg/kg DW, and that of cyanidin-based anthocyanins ranged from 943 to 3962 mg/kg DW. In each cultivar, peonidin-based anthocyanins showed much higher contents than cyanidin-based anthocyanins, except for the ‘Borami’ cultivar. The total peonidin-based anthocyanin content in the ‘Sinjami’ cultivar was approximately three-fold higher than the total cyanidin-based anthocyanin content. The total pelargonidin-based anthocyanin content was represented only in the outer and inner layer of the ‘Borami’ cultivar, ranging from 1242 to 2181 mg/kg DW. Among identified anthocyanins, 3-sophoroside-5-glucoside derivatives of peonidin and cyanidin, acylated with caffeic, ferulic, and *p*-hydroxybenzoic acids, predominated the anthocyanin composition of purple sweet potato. Relative proportions of cyanidin-, peonidin-, and pelargonidin-based anthocyanins in the ‘Borami’ cultivar were 1:6:1 in the outer layer and 1:7:1 in the inner layer. The ratio of cyanidin-, peonidin-, and pelargonidin-based anthocyanins was reported as 1:3:2 in the ‘Borami’ cultivar (980, 2810, and 2170 mg/kg DW, respectively) [22].

Anthocyanidin stability depends on the number of hydroxyl or methoxyl groups bonded with the B ring of aglycone. Their presence can decrease the aglycone stability; therefore, pelargonidin could contribute to higher stability than cyanidin and peonidin [34]. In a previous study, peonidin acylated with two *p*-coumaric acids showed higher stability than cyanidin acylated with two *p*-coumaric acids [35]. However, as mentioned above, anthocyanin stability can be further affected by the degree of acylation with phenolic acids. Therefore, it is necessary to study the relationship between changes in anthocyanin stability with the type of aglycone and the degree of acylation further.

In the outer and inner layers of all five cultivars, the most predominant anthocyanin component was peonidin 3-caffeoyl-*p*-hydroxybenzoyl sophoroside-5-glucoside (peak 17), as reported by previous studies [21,31]. The most abundant anthocyanin component in the ‘Jami’ cultivar was cyanidin 3-caffeoyl-*p*-hydroxybenzoyl sophoroside-5-glucoside, expressed as mg cyanidin-3-glucoside equivalent/100 g DW purple sweet potato [22]. To our knowledge, this is the first study to report the anthocyanin composition of the ‘Danjami’ cultivar.

### 3.3. Non-Anthocyanin Phenolic Composition of Purple Sweet Potato Cultivars

Table 3 shows the non-anthocyanin phenolic content in purple sweet potato cultivars analyzed using UHPLC-(ESI)-QqQ. The UHPLC-(ESI)-QqQ extracted ion chromatograms of non-anthocyanin phenolic compounds are shown in Figure S2. A total of 18 phenolic compounds belonging to hydroxycinnamic acid and flavonol classes were quantified in the MRM mode. Among the phenolic classes evaluated, chlorogenic acid (3-*O*-caffeoylquinic acid), two caffeoylquinic acid isomers, and five dicaffeoylquinic acid isomers were the predominant phenolic compounds in both outer and inner layers of the purple sweet cultivars.

In all cultivars, the major non-anthocyanin phenolic compound was caffeoylquinic acid isomer 2, followed by chlorogenic acid, regardless of the number of layers. In the outer layer, caffeoylquinic acid isomer 2 ranged from 30,433 to 86,062 mg/kg DW, and chlorogenic acid ranged from 12,818 to 20,650 mg/kg DW. In the inner layer, caffeoylquinic acid isomer 2 ranged from 24,029 to 56,801 mg/kg DW and chlorogenic acid from 6714 to 13,310 mg/kg DW. Chlorogenic acid content was significantly higher in the outer layer than in the inner layer in all cultivars (*p* < 0.05). ‘Jami’ contained a higher chlorogenic acid content (20,650 and 13,310 mg/kg DW for the outer and inner layer, respectively) than the other cultivars (*p* < 0.05), as with a previous study reporting higher chlorogenic acid concentration of ‘Jami’ than that of ‘Danjami’, ‘Sinjami’, and ‘Yeonjami’ [36]. ‘Danjami’ had significantly higher caffeoylquinic acid isomer 2 content (86,062 and 56,801 mg/kg DW for the outer and inner layer, respectively) than the other cultivars (*p* < 0.05).

Hydroxycinnamic acid derivatives consisted of *p*-coumaric, caffeic, *trans*-ferulic, and *cis*-ferulic acids and showed significantly higher concentrations in the outer layer than in the inner layer regardless of the cultivar (*p* < 0.05). The most abundant hydroxycinnamic acid derivative was caffeic acid ranging from 124 to 478 mg/kg DW in the outer layer and from 44 to 70 mg/kg DW in the inner layer. The highest concentration of caffeic acid was in the outer layer of ‘Danjami’ (478 mg/kg DW), corresponding to a previous study [36].

The predominant quercetin derivative was quercetin-*O*-diglucoside, ranging from 6 to 30 mg/kg DW in the outer layer and from 6 to 27 mg/kg DW in the inner layer. ‘Jami’ had significantly higher quercetin-*O*-diglucoside content (30 and 27 mg/kg DW for outer and inner layers, respectively) than the other cultivars (*p* < 0.05). Quercetin 3-*O-*galactoside was only quantified in the outer layers of ‘Sinjami’ (1 mg/kg DW) but was not detected in ‘Borami’ and ‘Danjami’. To the best of our knowledge, this is the first study to quantify quercetin derivatives in Korean purple sweet potato cultivars.

### 3.4. Total Phenolic Content and Antioxidant Activity

**Figure 2:**
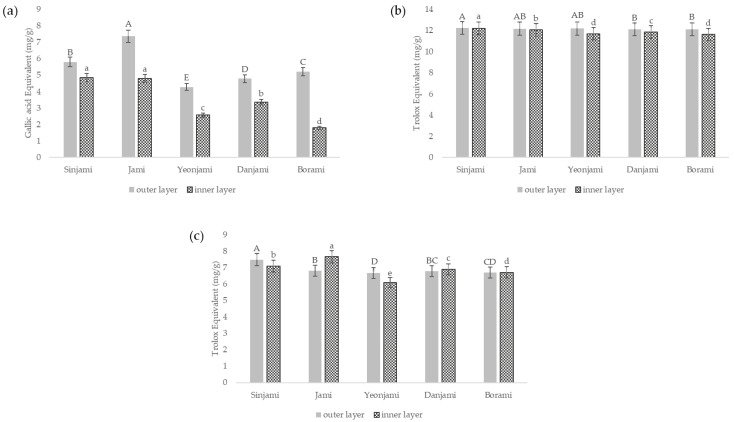
(**a**) Total phenolic content, (**b**) antioxidant activity measured by 2,2-diphenyl-1-picrylhydrazyl (DPPH) assay, and (**c**) antioxidant activity measured by performing 2,2′-azino-bis (3-ethylbenzothiazoline 6-sulphonic acid) (ABTS) assay of outer and inner layers of five purple sweet potato cultivars. Values designated with different uppercase and lowercase letters (A–D, a–d) differed significantly for the outer and inner layers, respectively (*p* < 0.05).

Total phenolic content (TPC) and antioxidant activity (determined by DPPH and ABTS assays) of the five cultivars are presented in Figure 2. The TPC value ranged from 1.80 to 7.37 mg GAE/g DW. DPPH radical scavenging activity ranged from 11.63 to 12.23 mg TEAC/g DW, and the ABTS radical scavenging activity ranged from 6.10 to 7.66 mg TEAC/g DW. In a previous study, TPC and DPPH values in the flesh sample of a Chinese purple sweet potato cultivar ranged from 1.83 to 13.85 mg GAE/g DW and 1.90 to 14.54 TEAC/g DW, respectively [37]. In this study, all purple sweet potato cultivars showed similar TPC and DPPH values as the previous study. Regarding varietal difference, the whole tuber of ‘Jami’ showed the highest DPPH and ABTS values (36.29 and 62.20 mg TEAC/g extract residue), followed by that of ‘Sinjami’, ‘Danjami’ and ‘Yeonjami’ [35]. This study did not compare the antioxidant activities of the outer and inner layers; therefore, the contents could not be directly compared. The outer layers showed higher TPC values than the inner layers, regardless of the cultivar (*p* < 0.05). The outer layer of ‘Jami’ had the highest TPC value (7.37 mg GAE/g DW), and the inner layer of ‘Jami’ and ‘Sinjami’ showed significantly higher TPC value than the other cultivars (*p* < 0.05). This study showed higher DPPH values in the outer layer of ‘Sinjami’, ‘Jami’, and ‘Yeonjami’ than in those of the other cultivars. DPPH values of the inner layer were the highest in ‘Sinjami’ (12.21 mg TEAC/g DW) and the lowest in ‘Borami’ (11.63 mg TEAC/g DW), similar to the anthocyanin content, whereas the highest ABTS value was in the inner layer of ‘Jami’ (7.66 mg TEAC/g DW) and the lowest in ‘Yeonjami’ (6.10 mg TEAC/g DW). The higher TPC and similar antioxidant activity (DPPH and ABTS values) in the outer layer than in the inner layer suggests the newly identified value of the outer layer as a rich source of phenolic compounds and antioxidants.

### 3.5. Principal Component Analysis and Heatmap of Purple Sweet Potato Cultivars

**Figure 3:**
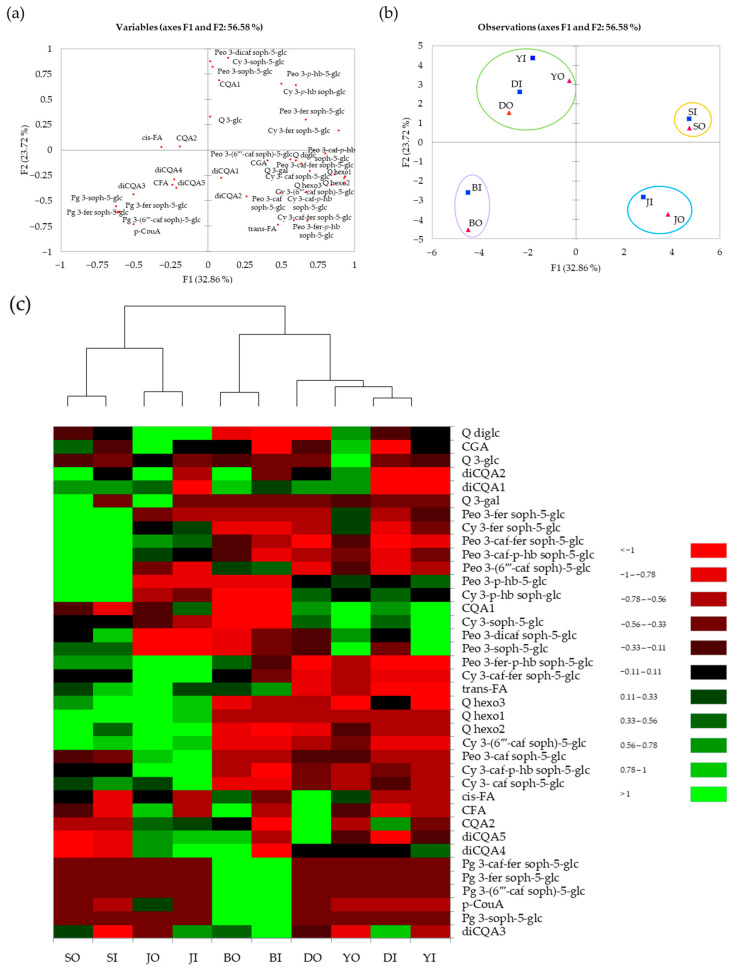

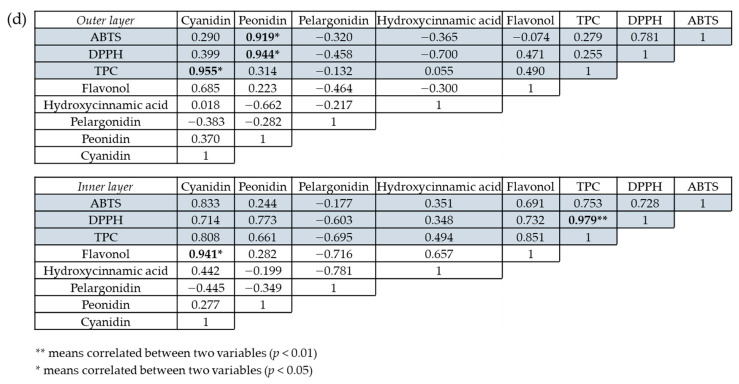
Principal component analysis (PCA) loading plot (**a**), PCA score plot (**b**), and heatmap (**c**) for visualization of varietal comparisons in different tissues (i.e., inner and outer layers) of the five purple sweet potato cultivars (S, Sinjami; D, Danjami; Y, Yeonjami; J, Jami; B, Borami; O, Outer layer; I, Inner layer) based on anthocyanin and non-anthocyanin phenolic compositions. (**d**) Pearson’s correlation among anthocyanins, non-anthocyanin phenolic compounds, total phenolic content, and antioxidant activities (ABTS and DPPH). Bold mean significance.

Figure 3a–c show the visualized results of PCA and heatmap based on anthocyanin composition and antioxidant activity in the five purple sweet potato cultivars. In Figure 3a,b, the first two principal components (PC1 and PC2) explained 56.58% of the total variables, with values of 32.86% and 23.72% for PC1 and PC2, respectively. The five cultivars were well-separated into four clusters (cluster 1, ‘Sinjami’ (SO, SI); cluster 2, ‘Jami’ (JO, JI); cluster 3, ‘Borami’ (BO, BI); and cluster 4, ‘Yeonjami’ (YO, YI) and ‘Danjami’ (DO, DI). The outer (O) and inner (I) layers of each cultivar were classified under the same cluster. Most of the di-acylated anthocyanins, chlorogenic acid, *trans*-ferulic acid, and quercetin hexosides were correlated with ‘Jami’, whereas ‘Sinjami’ correlated with mono-acylated anthocyanins. Cluster 3 samples were highly correlated with pelargonidin-based anthocyanins, caffeic acid, and *p*-coumaric acid.

Figure 3c presents a heatmap of identified anthocyanins and non-anthocyanin phenolic compounds in purple sweet potato samples. The data showed that the ‘Sinjami’ and ‘Jami’ cultivar contained higher cyanidin- and peonidin-based anthocyanin contents than the other cultivars. In addition, the two cultivars showed higher antioxidant activities (DPPH and ABTS). Among the five cultivars, ‘Borami’ was the only cultivar that exhibited high levels of pelargonidin-based anthocyanins. The ‘Yeonjami’ cultivar had high levels of non-acylated anthocyanins (i.e., cyanidin 3-sophoroside-5-glucoside and peonidin 3-sophoroside-5-glucoside). The ‘Jami’ cultivar showed higher levels of most quercetin derivatives than other cultivars.

Figure 3d displays Pearson’s correlation among anthocyanin contents, non-anthocyanin phenolic compound contents, TPC, and antioxidant activities (DPPH and ABTS). For simplification of the data presentation, the identified 20 individual anthocyanin compounds were grouped into three categories based on their aglycone (i.e., cyanidin, peonidin, and pelargonidin), and non-anthocyanin phenolic compounds were grouped into two categories (i.e., hydroxycinnamic acid and flavonol). Pearson’s correlations among individual phenolic compounds, total phenolic content (TPC), and antioxidant activities (ABTS and DPPH) of the outer and inner layers are shown in Table S2a–d. In the outer layer, peonidin content was positively correlated with antioxidant activities (DPPH and ABTS) (*p* < 0.05), and cyanidin contents positively correlated with only TPC (*p* < 0.05). Previous studies also showed a positive correlation between anthocyanin contents and antioxidant activities [38,39]. In the inner layer, cyanidin contents showed a significantly high correlation with flavonol contents (*p* < 0.05), with TPC positively correlated with DPPH activity (*p* < 0.05).

## 4. Conclusions

The composition of anthocyanin and non-anthocyanin phenolic compounds, TPC, and antioxidant activities (DPPH and ABTS) of five Korean purple sweet potato cultivars were investigated. Twenty anthocyanins (nine peonidin, seven cyanidin, and four pelargonidin derivatives) were tentatively identified using UHPLC-(ESI)-qTOF and UHPLC-Ion trap-MS. Anthocyanins in purple sweet potato are non-, mono-, or di-acylated derivatives of ferulic, caffeic, and *p*-hydroxybenzoic acids with the basic structure of peonidin 3-sophoroside-5-glucoside, cyanidin 3-sophoroside-5-glucoside, and pelargonidin 3-sophoroside-5-glucoside. In this study, pelargonidin-based anthocyanins were found only in ‘Borami’. Quantification of individual anthocyanins was performed using HPLC-DAD, and peonidin 3-caffeoyl-*p*-hydroxybenzoyl sophoroside-5-glucoside was identified as the major anthocyanin. Overall, the outer layers of the five purple sweet potato cultivars had higher anthocyanin contents than the inner layers, and the outer layer of ‘Sinjami’ showed the highest total anthocyanin concentration (30,446 mg/kg DW), followed by ‘Jami’, ‘Yeonjami’, ‘Borami’ and ‘Danjami’. Notably, di-acylated anthocyanins accounted for more than 90% of anthocyanins in ‘Jami’ and ‘Borami’ and 72–77% in ‘Sinjami’, ‘Danjami’ and ‘Yeonjami’ (*p* < 0.05). Additionally, 18 non-anthocyanin phenolic compounds were determined using UHPLC-(ESI)-QqQ. The major non-anthocyanin phenolic compound was caffeoylquinic acid isomer 2 (24,029–86,062 mg/kg DW), followed by chlorogenic acid (6714–20,650 mg/kg DW) in all cultivars. TPC and DPPH radical scavenging activities were higher in the outer layer than in the inner layer, as with the anthocyanin content.

This study is the first systemic investigation of anthocyanin and non-anthocyanin phenolic compounds and antioxidant activities in various Korean purple sweet potato cultivars and can provide a new health value for the outer layer of purple sweet potato.

## Figures and Tables

**Table 1:** Identified anthocyanins in purple sweet potato using an ultra-high-performance liquid chromatography-positive electrospray-time-of-flight mass spectrometer (UHPLC-(ESI)-qTOF MS).

Peak ^2^	Compound	Theoretical Mass (*m*/*z*)	Fragment Ion (*m*/*z*) ^3^	Mass Error (ppm) ^4^	Sinjami	Jami	Yeonjami	Danjami	Borami
1	Cy 3-soph-5-glc	773.2135	611/449/287	1.4	O ^1^	O	O	O	O
2	Pg 3-soph-5-glc	757.2186	595/433/271	1.8	X	X	O	X	O
3	Peo 3-soph-5-glc	787.2291	625/463/301	2.5	O	O	O	O	O
4	Cy 3-*p*-hb soph-glc	893.2346	731/449/287	2.6	O	O	O	O	O
5	Cy 3-(6‴-caf soph)-5-glc	935.2452	773/449/287	2.8	O	X	X	X	X
6	Peo 3-*p*- hb soph-5-glc	907.2503	745/463/301	2.5	O	O	O	O	O
7	Peo 3-(6‴-caf soph)-5-glc	949.2608	787/463/301	2.9	O	O	O	O	O
8	Cy 3-fer soph-5-glc	949.2608	787/449/287	2.4	O	O	O	O	X
9	Pg 3-(6‴-caf soph)-5-glc	919.2503	757/433/271	1.0	X	X	X	X	O
10	Peo 3-fer soph-5-glc	963.2765	801/463/301	2.6	O	O	O	O	O
11	Pg 3-fer soph-5-glc	933.2659	771/433/271	−6.3	X	X	X	X	O
12	Cy 3-caf soph-5-glc	935.2452	773/449/287	3.6	O	O	O	O	O
13	Cy 3-caf-*p*-hb soph-5-glc	1055.2663	893/449/287	2.9	O	O	O	O	O
14	Peo 3-caf soph-5-glc	949.2608	787/463/301	3.3	O	O	X	X	X
15	Cy 3-caf-fer soph-5-glc	1111.2925	949/449/287	4.0	O	O	O	O	O
16	Peo 3-dicaf soph-5-glc	1111.2925	949/463/301	4.0	O	O	O	O	O
17	Peo 3-caf-*p*-hb soph-5-glc	1069.2820	907/463/301	3.1	O	O	O	O	O
18	Peo 3-caf-fer soph-5-glc	1125.3082	963/463/301	3.2	O	O	O	O	O
19	Pg 3-caf-fer soph-5-glc	1095.2976	933/433/271	3.0	X	X	O	X	O
20	Peo 3-fer-*p*-hb soph-5-glc	1083.2976	921/463/301	3.9	O	O	X	X	X

^1^ O and X mean detected and not detected, respectively. ^2^ Peak numbers are the same as the peaks shown in the chromatograms in Figure S1. ^3^ Fragment ions were determined using UHPLC-Iontrap MS. ^4^ Mass error was calculated by comparing predicted *m*/*z* and measured *m*/*z* using UHPLC-(ESI)-qTOF MS. Abbreviations: Cy, cyanidin; Pg, pelargonidin; Peo, peonidin; p-hb, p-hydroxybenzoic acid; caf, caffeic acid; fer, ferulic acid; soph, sophoroside; glc, glucoside.

**Table 2:** Anthocyanin content in purple sweet potato cultivars determined using a high-performance liquid chromatography-photodiode array detector (HPLC-DAD).

		Anthocyanin Content (mg/kg DW)									
		Outer Layer					Inner Layer				
Peak ^1^	Compound	SO	DO	YO	JO	BO	SI	DI	YI	JI	BI
1	Cy 3-soph-5-glc	66 ± 5 ^C^	82 ± 6 ^B^	114 ± 6 ^A^	54± 2 ***^D^	9 ± 1 *^E^	67 ± 1 ^c^	81 ± 2 ^b^	124 ± 2 ^a^	35 ± 2 ^d^	11 ± 1 ^e^
2	Pg 3-soph-5-glc	N.D.	N.D.	N.D.	N.D.	70 ± 11 *	N.D.	N.D.	N.D.	N.D.	100 ± 9
3	Peo 3-soph-5-glc	368 ± 27 ^B^	184 ± 12 *^C^	688 ± 37 ^A^	4 ± 1 ^E^	73 ± 6 **^D^	370 ± 14 ^b^	181 ± 17 ^c^	635 ± 32 ^a^	N.D.	145 ± 11 ^d^
4	Cy 3-*p*-hb soph-glc	488 ± 14 **^A^	280 ± 13 **^B^	234 ± 17 ^C^	135 ± 3 **^D^	15 ± 1 ***^E^	386 ± 26 ^a^	305 ± 5 ^b^	228 ± 5 ^c^	161 ± 6 ^d^	6 ± 1 ^e^
5	Cy 3-(6‴-caf soph)-5-glc	72 ± 3 *^B^	20 ± 2 ^CD^	26 ± 1 ***^C^	82 ± 7 *^A^	14 ± 1 ^E^	59 ± 3 ^a^	10 ± 1 ^bc^	10 ± 1 ^c^	60 ± 3 ^a^	14 ± 1 ^b^
6	Peo 3-*p*-hb-5-glc	2673 ± 88 *^A^	1123 ± 67.7 ^C^	1410 ± 27 ***^B^	200 ± 12 **^D^	112 ± 9 **^D^	3177 ± 200 ^a^	1193 ± 19 ^c^	1613 ± 16 ^b^	125 ± 4 ^d^	170 ± 12 ^d^
7	Peo 3-(6‴-caf soph)-5-glc	421 ± 21.4 ^A^	33 ± 2 ^E^	120 ± 9 **^C^	87 ± 6 **^D^	215 ± 15 ^B^	423 ± 15 ^a^	33 ± 2 ^d^	60 ± 8 ^c^	45 ± 4 ^cd^	247 ± 13 ^b^
8	Cy 3-fer soph-5-glc	512 ± 17 ^A^	62 ± 5 **^D^	225 ± 7 ***^B^	196 ± 12 ^C^	N.D.	466 ± 26 ^a^	29 ± 1 ^d^	102 ± 4 ^c^	205 ± 11 ^b^	N.D.
9	Pg 3-(6‴-caf soph)-5-glc	N.D.	N.D.	N.D.	N.D.	15 ± 1 **^A^	N.D.	N.D.	N.D.	N.D.	11 ± 1 ^a^
10	Peo 3-fer soph-5-glc	2979 ± 220 ^A^	129 ± 8 ***^C^	1074 ± 27 ***^B^	184 ± 11 ***^C^	133 ± 8 ^C^	3064 ± 206 ^a^	54 ± 5 ^c^	525 ± 19 ^b^	83 ± 7 ^c^	117 ± 10 ^c^
11	Pg 3-fer soph-5-glc	N.D.	N.D.	N.D.	N.D.	107 ± 15 *^A^	N.D.	N.D.	N.D.	N.D.	64 ± 9
12	Cy 3-caf soph-5-glc	72 ± 2 **^A^	33 ± 2 ^C^	10 ± 1 ***^D^	66 ± 3 ***^B^	N.D.	95 ± 4.7 ^b^	40 ± 2 ^c^	20 ± 1 ^d^	190 ± 7 ^a^	4 ± 1 ^e^
13	Cy 3-caf-p-hb soph-5-glc	1866 ± 101 *^B^	1232 ± 119 ^C^	892 ± 16 ^D^	3928 ± 151 *^A^	796 ± 60 ***^D^	1663 ± 37 ^b^	1179 ± 34 ^c^	965 ± 43 ^c^	4572 ± 300 ^a^	336 ± 10 ^d^
14	Peo 3-caf soph-5-glc	7 ± 1 **^BC^	5 ± 1 ***^C^	8 ± 1 ***^B^	28 ± 2 ***^A^	N.D.	3 ± 1 ^b^	1 ± 1 ^c^	N.D.	54 ± 2 ^a^	N.D.
15	Cy 3-caf-fer soph-5-glc	883 ± 33 ^B^	161 ± 11 ***^D^	368 ± 5 ***^C^	2537 ± 104 ^A^	894 ± 74 **^B^	936 ± 33 ^b^	62.1 ± 5 ^e^	188 ± 9 ^d^	2490 ± 131 ^a^	572 ± 30 ^c^
16	Peo 3-dicaf soph-5-glc	677 ± 17 **^B^	598 ± 55 ^C^	931 ± 17 ***^A^	194 ± 18 *^E^	256 ± 20 ***^D^	1050 ± 65 ^b^	672 ± 54 ^c^	1470 ± 71 ^a^	230 ± 7 ^e^	459 ± 10 ^d^
17	Peo 3-caf-*p*-hb soph-5-glc	12,366 ± 121 **^A^	3974 ± 326 ^E^	4996 ± 129 ^D^	7414 ± 269 ^B^	6059 ± 491 **^C^	14,832 ± 529 ^a^	3464 ± 110 ^d^	4911 ± 300 ^c^	6612 ± 436 ^b^	3790 ± 130 ^d^
18	Peo 3-caf-fer soph-5-glc	6837 ± 173 *^A^	495 ± 24 ***^D^	2867 ± 80 ***^C^	5060 ± 216 *^B^	2848 ± 269 **^C^	7831 ± 400 ^a^	158 ± 4 ^e^	1145 ± 59 ^d^	4283 ± 273 ^b^	1639 ± 58 ^c^
19	Pg 3-caf-fer soph-5-glc	N.D.	N.D.	N.D.	N.D.	1988 ± 119 ***^A^	N.D.	N.D.	N.D.	N.D.	1066 ± 46 ^a^
20	Peo 3-fer-*p*-hb soph-5-glc	152 ± 10 ^B^	4 ± 1 **^E^	40 ± 2 ***^D^	188 ± 19 ^A^	122 ± 7 **^C^	141.4 ± 6 ^b^	10.0 ± 1 ^d^	19 ± 1 ^d^	201 ± 13 ^a^	78 ± 6 ^c^
Total anthocyanin		30,446 ± 721 *^A^	8421 ± 633 ^D^	14010 ± 107 **^C^	20,365 ± 776 ^B^	13,732 ± 1037 **^C^	34,569 ± 1493 ^a^	7477 ± 214 ^d^	12,020 ± 523 ^c^	19,352 ± 1154 ^b^	8833 ± 247 ^d^
Non-acylated anthocyanin		435 ± 32 ^B^ (1%)	266 ± 17 ^C^ (3%)	802.9 ± 40 ^A^ (6%)	59 ± 1 ***^E^ (0%)	152 ± 7 **^D^ (1%)	437 ± 14 ^b^ (1%)	262 ± 16 ^c^ (4%)	759 ± 33 ^a^ (6%)	35 ± 1 ^d^ (0%)	257 ± 18 ^c^ (3%)
Mono-acylated anthocyanin		7227 ± 340 ^A^ (24%)	1681 ± 86 ^C^ (20%)	3110 ± 73 ***^B^ (22%)	952 ± 13 **^D^ (5%)	614 ± 34 ^E^ (4%)	7676 ± 467 ^a^ (22%)	1667 ± 28 ^c^ (22%)	2561 ± 34 ^b^ (21%)	872 ± 8 ^d^ (5%)	634 ± 22 ^d^ (7%)
Di-acylated anthocyanin		22,783 ± 407 *^A^ (75%)	6467 ± 532 *^E^ (77%)	10096 ± 194 **^D^ (72%)	19,324 ± 763 ^B^ (95%)	12,965 ± 1003 **^C^ (94%)	26,455 ± 1036 ^a^ (77%)	5546 ± 194 ^d^ (74%)	8699 ± 468 ^c^ (72%)	18,389 ± 1148 ^b^ (95%)	7941 ± 244 ^c^ (90%)
Cyanidin-based anthocyanin		3962 ± 129 ^B^	1871 ± 153 ^C^	1872 ± 44 **^C^	7001 ± 256 ^A^	1730 ± 131 **^C^	3675 ± 119 ^b^	1709 ± 36 ^c^	1639 ± 40 ^c^	7716 ± 444 ^a^	943 ± 34 ^d^
Peonidin-based anthocyanin		26,483 ± 612 *^A^	6544 ± 481 ^E^	12,137 ± 147 **^C^	13,335 ± 525 *^B^	9820 ± 780 **^D^	30,894 ± 1374 ^a^	5767 ± 178 ^c^	10,381 ± 484 ^b^	11,581 ± 721 ^b^	6647 ± 189 ^c^
Pelargonidin-based anthocyanin		N.D.	N.D.	N.D.	N.D.	2181 ± 139 **^A^	N.D.	N.D.	N.D.	N.D.	1242 ± 43 ^a^

Abbreviates: S, Sinjami; D, Danjami; Y, Yeonjami; J, Jami; B, Borami; O, Outer layer; I, Inner layer.; *, **, *** indicate significant differences between outer and inner layers in same cultivar at *p* < 0.05, *p* < 0.01 and *p* < 0.001, respectively. Contents in the table are expressed as the average content ± SD. Values designated with different uppercase and lowercase letters (A–E, a–e) differed significantly for the outer and inner layers, respectively (*p* < 0.05). ^1^ Peak numbers are the same as the peaks shown in the chromatograms of Figure S1. N.D.: not detected.

**Table 3:** Phenolic content in the outer and inner layers of five purple sweet potato cultivars by ultra-high-performance liquid chromatography-positive electrospray-triple quadrupole (UHPLC-(ESI)-QqQ) (mg/kg DW).

Polyphenols		Sinjami		Danjami		Yeonjami		Jami		Borami	
Peak ^1^	Compound	Outer	Inner	Outer	Inner	Outer	Inner	Outer	Inner	Outer	Inner
	*Hydroxycinnamic acids*										
g	*p*-Coumaric acid	N.Q.	N.Q.	N.Q.	N.Q.	N.Q.	N.Q.	N.Q.	N.Q.	1 ± 0 *^A^	1 ± 0 ^a^
f	Caffeic acid	143 ± 1 ***^D^	44 ± 0 ^e^	478 ± 8 ***^A^	46 ± 0 ^d^	124 ± 3 ***^E^	55 ± 1 ^c^	286 ± 2 ***^C^	65 ± 1 ^b^	318 ± 1 ***^B^	70 ± 0 ^a^
h	*trans*-Ferulic acid	5 ± 0 ***^B^	7 ± 0 ^a^	2 ± 0 ***^D^	N.Q.	3 ± 0 ***^C^	1 ± 0 ^c^	11 ± 1 ***^A^	5 ± 0 ^b^	5 ± 0 **^B^	7 ± 0 ^a^
m	*cis*-Ferulic acid	11 ± 0 ***^CD^	2 ± 0 ^c^	40.3 ± 3.4 **^A^	4 ± 0 ^b^	14 ± 0 ***^BC^	3 ± 0 ^b^	10 ± 1 ***^D^	3 ± 0 ^c^	16 ± 1 ***^B^	7 ± 1 ^a^
b	Caffeoylquinic acid isomer 1	5528 ± 2 ^C^	5378 ± 90 ^b^	5705 ± 44 ^B^	5702 ± 34 ^a^	5821 ± 82 ^A^	5862 ± 99 ^a^	5514 ± 13 ^C^	5662 ± 4 ^a^	5338 ± 13 ^D^	5150 ± 236 ^c^
o	Caffeoylquinic acid isomer 2	30,433 ± 1281 ^E^	31,178 ± 467 ^d^	86,062 ± 745 ***^A^	56,801 ± 395 ^a^	32,294 ± 436 ***^D^	35,635 ± 133 ^c^	52,319 ± 1027 **^B^	47,086 ± 348 ^b^	42,620 ± 145 ***^C^	24,029 ± 406 ^e^
a	Dicaffeoylquinic acid isomer 1	23 ± 0 ^BC^	24 ± 2 ^a^	23 ± 1 ***^BC^	N.D.	24 ± 2 **^AB^	N.D.	21 ± 1 ***^C^	N.D.	26 ± 0 ***^A^	19 ± 0 ^b^
c	Dicaffeoylquinic acid isomer 2	73 ± 4 **^A^	50 ± 3 ^a^	54 ± 5 **^C^	27 ± 2 ^d^	65 ± 2 ***^B^	25 ± 1 ^d^	73 ± 0 **^A^	38 ± 2 ^c^	75 ± 1 ***^A^	43 ± 2 ^b^
n	Dicaffeoylquinic acid isomer 3	412 ± 16 **^AB^	263 ± 3 ^d^	376 ± 11 **^BC^	454 ± 12 ^b^	334 ± 33 ^C^	352 ± 37 ^c^	367 ± 18 **^BC^	443 ± 18 ^b^	426 *^A^ ± 36 *^A^	531 ± 37 ^a^
p	Dicaffeoylquinic acid isomer 4	606 ± 13 ^C^	694 ± 45 ^c^	826 ± 47 ^B^	804 ± 84 ^b^	826 ± 15 ^B^	859 ± 73 ^b^	898 ± 56 ^AB^	973 ± 26 ^a^	960 ± 39 ***^A^	689 ± 12 ^c^
r	Dicaffeoylquinic acid isomer 5	38 ± 1 ^D^	39 ± 1 ^d^	81 ± 8 *^A^	37 ± 3 ^d^	52 ± 3^C^	51 ± 1 ^b^	63 ± 5 ^B^	67 ± 2 ^a^	68 ± 7 **^B^	43 ± 1 ^c^
e	Chlorogenic acid	14,739 ± 19 **^C^	12,831 ± 24 ^b^	12,818 ± 49 ***^E^	8360 ± 25 ^c^	17,244 ± 440 ***^B^	13,268 ± 49 ^a^	20,650 ± 103 ***^A^	13,310 ± 203 ^a^	13,258 ± 276 ***^D^	6714 ± 48 ^d^
	*Flavonols*										
i	Quercetin diglucoside	13 ± 0 *^C^	15 ± 1 ^b^	6 ± 1 ***^E^	15 ± 0 ^b^	21 ± 1 ***^B^	15 ± 1 ^b^	30 ± 1 *^A^	27 ± 1 ^a^	8 ± 0 **^D^	6 ± 0 ^c^
d	Quercetin hexoside 1	1 ± 0 ^A^	1 ± 0 ^a^	N.Q.	N.Q.	N.Q.	N.Q.	1 ± 0 ^A^	1 ± 0 ^a^	N.Q.	N.Q.
j	Quercetin hexoside 2	1 ± 0 *^B^	1 ± 0 ^a^	N.Q.	1 ± 0 ^a^	1 ± 0 *^B^	N.Q.	2 ± 0 *^A^	1 ± 0 ^a^	N.Q.	N.Q.
k	Quercetin 3-*O*-galactoside	1 ± 0 **^A^	N.D.	N.D.	N.D.	N.Q.	N.D.	N.Q.	N.D.	N.D.	N.D.
l	Quercetin 3-*O*-glucoside	1 ± 0 ***^B^	N.Q.	N.Q.	N.Q.	7 ± 0 ***^A^	1 ± 0 ^a^	1 ± 0 **^B^	N.Q.	1 ± 0 *^B^	N.Q.
q	Quercetin hexoside 3	1 ± 0 **^B^	2 ± 0 ^a^	N.Q.	1 ± 0 ^b^	N.D.	N.D.	2 ± 0 *^A^	1 ± 0 ^b^	N.Q.	N.Q.

^1^ Peak numbers are the same as the peaks shown in the chromatograms in Figure S2. Abbreviations: *, **, *** indicate significant differences between the outer and inner layers in the same cultivar at *p* < 0.05, *p* < 0.01, and *p* < 0.001, respectively. Contents in the table are expressed as the average content ± SD. Mean values followed by different lower case and capital letters indicate significant cultivar differences for the inner or outer layers, respectively, at *p* < 0.05. N.D.: not detected. N.Q.: not quantified with the calibration curve.

## Data Availability

Data is contained within the article or supplementary material.

## Supplementary Materials

The following are available online at https://www.mdpi.com/2076-3921/10/3/462/s1, Figure S1: Representative HPLC-DAD chromatograms of anthocyanins in purple sweet potato cultivars: (a) outer layer of Yeonjami, (b) inner layer of Yeonjami, (c) outer layer of Borami, (d) inner layer of Borami, Figure S2: Representative UHPLC-(ESI)-QqQ MS extracted ion chromatograms of non-anthocyanin phenolic compounds in the outer layer of Sinjami: (a) a, dicaffeoylquinic acid isomer 1; c, dicaffeoylquinic acid isomer 2; d, quercetin hexoside 1; f, caffeic acid; g, p-coumaric acid; h, trans-ferulic acid; i, quercetin diglucoside; j, quercetin hexoside 2; k, quercetin 3-O-galactoside; l, quercetin 3-O-glucoside; m, cis-ferulic acid; n, dicaffeoylquinic acid isomer 3; p, dicaffeoylquinic acid isomer 4; q, quercetin hexoside 3; r, dicaffeoylquinic acid isomer 5, (b) b, caffeoylquinic acid isomer 1; e, chlorogenic acid; o, caffeoylquinic acid isomer 2, Table S1: F- and *p*-values of all purple sweet potato samples for individual phenolic contents, total phenolic content (TPC), and antioxidant activity by multivariate ANOVA, Table S2: Pearson’s correlations among individual anthocyanin compounds, total phenolic content (TPC), and antioxidant activities (ABTS and DPPH) of the outer layer.
