# Supplementary material for: Phenolic Composition and Antioxidant Activity of Purple Sweet Potato (Ipomoea batatas (L.) Lam.): Varietal Comparisons and Physical Distribution
Source: Antioxidants (Basel). 2021 Mar 16;10(3):462. doi: 10.3390/antiox10030462 (PMC8000629; doi:10.3390/antiox10030462)
Supplement: Supplementary file 1 [file antioxidants-10-00462-s001.zip › Supplementary figure 1.docx]

**Supplementary Materials**


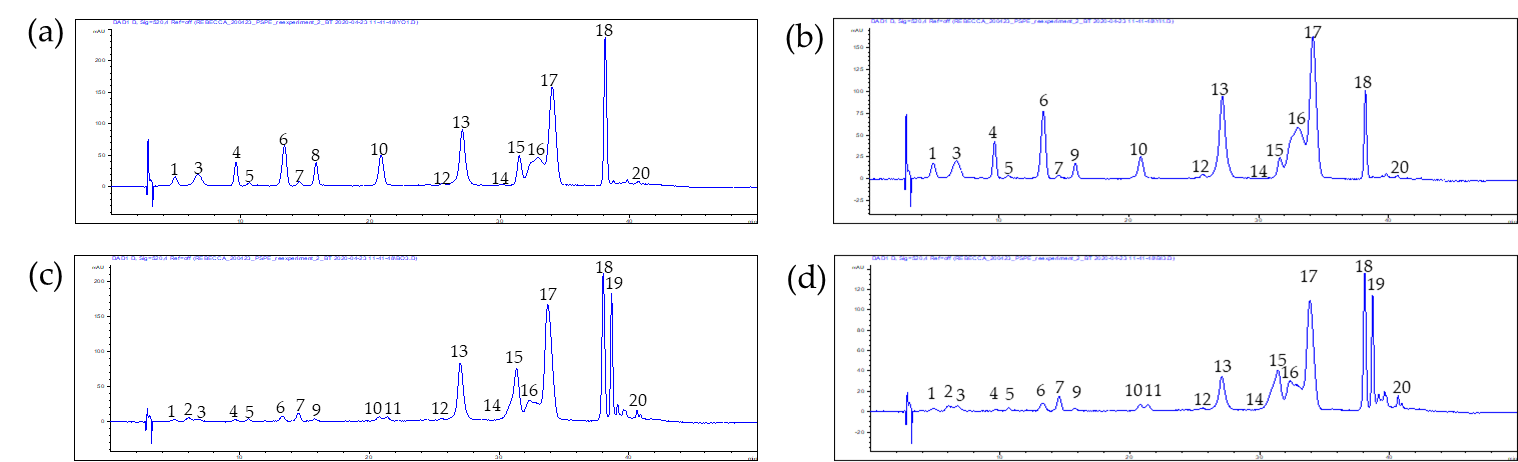


**Figure S1.** Representative HPLC-DAD chromatograms of anthocyanins in purple sweet potato cultivars: (a) outer layer of Yeonjami, (b) inner layer of Yeonjami, (c) outer layer of Borami, (d) inner layer of Borami.
