# Supplementary material for: Phenolic Composition and Antioxidant Activity of Purple Sweet Potato (Ipomoea batatas (L.) Lam.): Varietal Comparisons and Physical Distribution
Source: Antioxidants (Basel). 2021 Mar 16;10(3):462. doi: 10.3390/antiox10030462 (PMC8000629; doi:10.3390/antiox10030462)
Supplement: Supplementary file 1 [file antioxidants-10-00462-s001.zip › Supplementary figure 2.docx]

**Supplementary Materials**


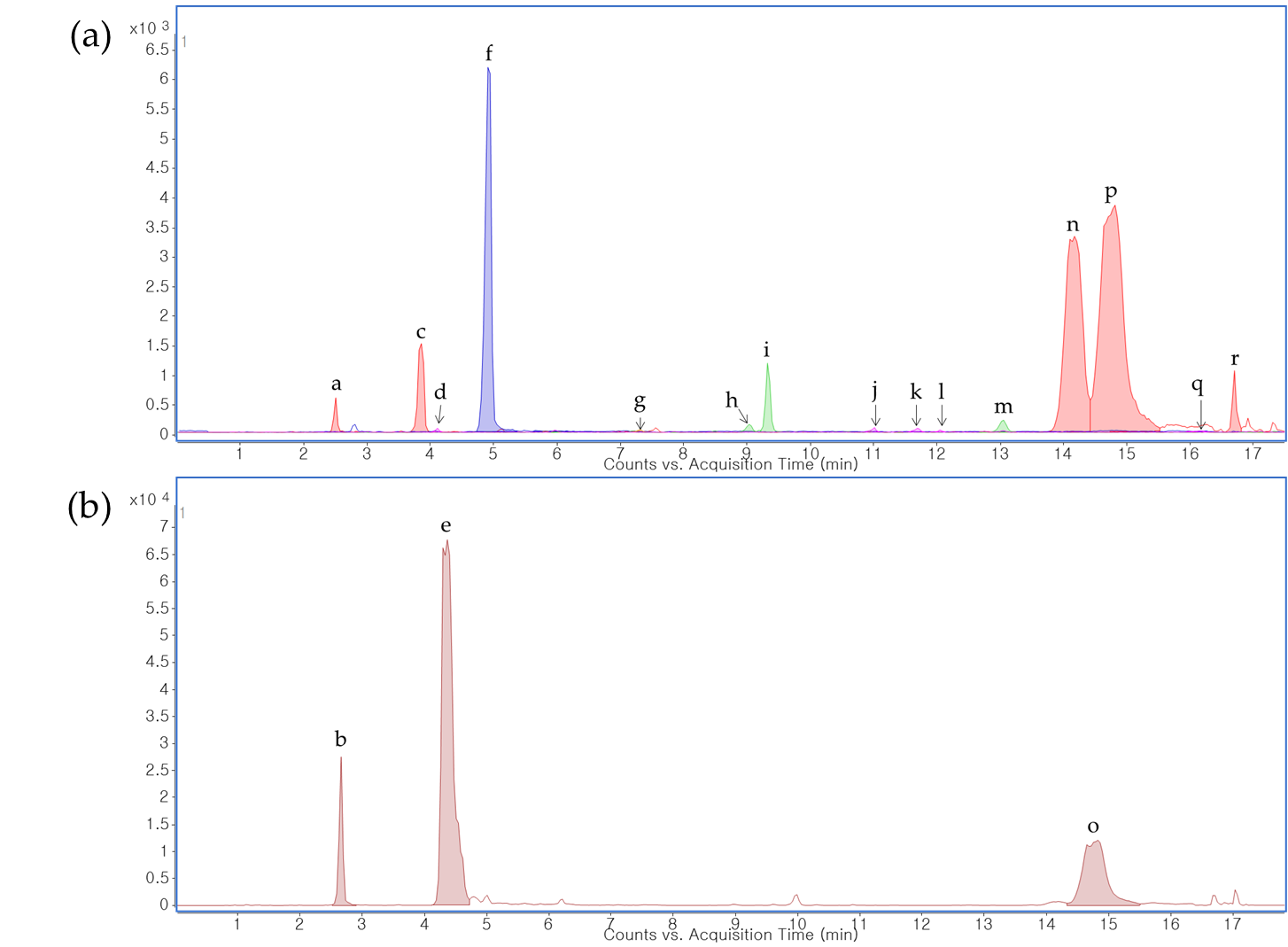


**Figure S2.** Representative UHPLC-(ESI)-QqQ MS extracted ion chromatograms of non-anthocyanin phenolic compounds in the outer layer of Sinjami: (a) a, dicaffeoylquinic acid isomer 1; c, dicaffeoylquinic acid isomer 2; d, quercetin hexoside 1; f, caffeic acid; g, *p*-coumaric acid; h, *trans*-ferulic acid; i, quercetin diglucoside; j, quercetin hexoside 2; k, quercetin 3-*O-*galactoside; l, quercetin 3-*O*-glucoside; m, *cis*-ferulic acid; n, dicaffeoylquinic acid isomer 3; p, dicaffeoylquinic acid isomer 4; q, quercetin hexoside 3; r, dicaffeoylquinic acid isomer 5, (b) b, caffeoylquinic acid isomer 1; e, chlorogenic acid; o, caffeoylquinic acid isomer 2.
