# Supplementary material for: Phenolic Composition and Antioxidant Activity of Purple Sweet Potato (Ipomoea batatas (L.) Lam.): Varietal Comparisons and Physical Distribution
Source: Antioxidants (Basel). 2021 Mar 16;10(3):462. doi: 10.3390/antiox10030462 (PMC8000629; doi:10.3390/antiox10030462)
Supplement: Supplementary file 1 [file antioxidants-10-00462-s001.zip › Supplementary Table 1.docx]

**Table S1.** F- and *p*-values of all purple sweet potato samples for individual phenolic contents, total phenolic content (TPC), and antioxidant activity by multivariate ANOVA.

|  | **F-value** | | | ***p-*value** | | | |
| --- | --- | --- | --- | --- | --- | --- | --- |
| Compound | **Cultivar** | **Layer** | **Cultivar x Layer** | **Cultivar** | | **Layer** | **Cultivar x Layer** |
| Cy 3-soph-5-glc | 746.6 | 1.8 | 13.2 | ****** | NS | | ****** |
| Pg 3-soph-5-glc | 399.7 | 12.0 | 12.0 | ****** | ****** | | ****** |
| Peo 3-soph-5-glc | 997.5 | 0.1 | 7.5 | ****** | NS | | ****** |
| Cy 3-*p*-hb soph-glc | 1012.3 | 8.7 | 27.4 | ****** | ****** | | ****** |
| Cy 3-(6″′-caf soph)-5-glc | 512.7 | 109.2 | 9.6 | ****** | ****** | | ****** |
| Peo 3-*p*-hb-5-glc | 1449.2 | 31.7 | 13.3 | ****** | ****** | | ****** |
| Peo 3-(6″′-caf soph)-5-glc | 1153.0 | 10.2 | 15.1 | ****** | ****** | | ****** |
| Cy 3-fer soph-5-glc | 1594.8 | 80.7 | 29.9 | ****** | ****** | | ****** |
| Pg 3-(6″′-caf soph)-5-glc | 1430.0 | 26.4 | 26.4 | ****** | ****** | | ****** |
| Peo 3-fer soph-5-glc | 1023.7 | 13.9 | 9.6 | ****** | ****** | | ****** |
| Pg 3-fer soph-5-glc | 271.8 | 17.3 | 17.3 | ****** | ****** | | ****** |
| Cy 3- caf soph-5-glc | 1461.0 | 757.8 | 352.0 | ****** | ****** | | ****** |
| Cy 3-caf-*p*-hb soph-5-glc | 884.4 | 0.0 | 17.3 | ****** | NS | | ****** |
| Peo 3-caf soph-5-glc | 1038.2 | 11.7 | 177.7 | ****** | ****** | | ****** |
| Cy 3-caf-fer soph-5-glc | 1468.7 | 28.6 | 8.1 | ****** | ****** | | ****** |
| Peo 3-dicaf soph-5-glc | 554.1 | 265.9 | 39.3 | ****** | ****** | | ****** |
| Peo 3-caf-*p*-hb soph-5-glc | 902.5 | 4.2 | 42.7 | ****** | NS | | ****** |
| Peo 3-caf-fer soph-5-glc | 1122.0 | 70.2 | 40.2 | ****** | ****** | | ****** |
| Pg 3-caf-fer soph-5-glc | 1692.5 | 154.0 | 154.0 | ****** | ****** | | ****** |
| Peo 3-fer-*p*-hb soph-5-glc | 445.5 | 12.1 | 9.2 | ****** | ****** | | ****** |
| Total anthocyanin | 867.0 | 10.2 | 24.2 | ****** | ****** | | ****** |
| Non-acylated anthocyanin | 934.6 | 0.6 | 9.8 | ****** | NS | | ****** |
| Mono-acylated anthocyanin | 1330.7 | 0.3 | 5.4 | ****** | NS | | ****** |
| Di-acylated anthocyanin | 733.0 | 13.3 | 29.9 | ****** | ****** | | ****** |
| Cyanidin-based anthocyanin | 1109.7 | 5.0 | 13.0 | ****** | ***** | | ****** |
| Peonidin-based anthocyanin | 1129.7 | 6.6 | 30.5 | ****** | ***** | | ****** |
| Pelargonidin-based anthocyanin | 1646.8 | 123.9 | 123.9 | ****** | ****** | | ****** |
| TPC | 435.6 | 1592.2 | 75.3 | ****** | ****** | | ****** |
| Antioxidant activity (DPPH assay) | 7.1 | 164.6 | 9.3 | ****** | ****** | | ****** |
| Antioxidant activity (ABTS assay) | 259.58 | 0.1 | 135.398 | ****** | NS | | ****** |
| Caffeic acid | 3624.9 | 39047.2 | 3537.6 | ****** | ****** | | ****** |
| Chlorogenic acid | 1627.4 | 5327.3 | 214.7 | ****** | ****** | | ****** |
| Caffeoylquinic acid isomer 1 | 37.8 | 0.8 | 3.4 | ****** | NS | | ***** |
| Caffeoylquinic acid isomer 2 | 4253.7 | 1740.4 | 689.3 | ****** | ****** | | ****** |
| Dicaffeoylquinic acid isomer 1 | 1058.1 | 6978.3 | 842.8 | ****** | ****** | | ****** |
| Dicaffeoylquinic acid isomer 2 | 75.6 | 900.3 | 27.2 | ****** | ****** | | ****** |
| Dicaffeoylquinic acid isomer 3 | 32.6 | 8.1 | 25.7 | ****** | ***** | | ****** |
| Dicaffeoylquinic acid isomer 4 | 28.2 | 1.3 | 14.4 | ****** | NS | | ****** |
| Dicaffeoylquinic acid isomer 5 | 39.8 | 87.0 | 44.8 | ****** | ****** | | ****** |
| *p*-Coumaric acid | 912.0 | 242.0 | 102.0 | ****** | ****** | | ****** |
| *trans*-Ferulic acid | 639.9 | 130.4 | 162.6 | ****** | ****** | | ****** |
| *cis*-Ferulic acid | 168.8 | 1082.9 | 150.6 | ****** | ****** | | ****** |
| Quercetin diglucoside | 1037.1 | 0.8 | 125.1 | ****** | NS | | ****** |
| Quercetin hexoside 1 | 513.8 | 0.3 | 11.4 | ****** | NS | | ****** |
| Quercetin hexoside 2 | 251.4 | 41.9 | 9.7 | ****** | ****** | | ****** |
| Quercetin 3-*O*-galactoside | 326.6 | 952.1 | 326.6 | ****** | ****** | | ****** |
| Quercetin 3-*O*-glucoside | 4732.6 | 6643.6 | 3794.4 | ****** | ****** | | ****** |
| Quercetin hexoside 3 | 690.4 | 35.6 | 46.2 | ****** | ****** | | ****** |

NS, *, and ** indicate *p* > 0.05 (not significantly different), *p* < 0.05, and *p* < 0. 1, respectively.
