# Supplementary material for: Phenolic Composition and Antioxidant Activity of Purple Sweet Potato (Ipomoea batatas (L.) Lam.): Varietal Comparisons and Physical Distribution
Source: Antioxidants (Basel). 2021 Mar 16;10(3):462. doi: 10.3390/antiox10030462 (PMC8000629; doi:10.3390/antiox10030462)
Supplement: Supplementary file 1 [file antioxidants-10-00462-s001.zip › Supplementary Table 2.docx]

**Table S2(A).** Pearson’s correlations among individual anthocyanin compounds, total phenolic content (TPC), and antioxidant activities (ABTS and DPPH) of the outer layer.

| *Outer layer* | 1^1^ | 2 | 3 | 4 | 5 | 6 | 7 | 8 | 9 | 10 | 11 | 12 | 13 | 14 | 15 | 16 | 17 | 18 | 19 | 20 | TPC | DPPH | ABTS |
| --- | --- | --- | --- | --- | --- | --- | --- | --- | --- | --- | --- | --- | --- | --- | --- | --- | --- | --- | --- | --- | --- | --- | --- |
| ABTS | -0.141 | -0.170 | 0.028 | 0.745 | 0.551 | 0.741 | **0.892^*^** | 0.813 | -0.170 | 0.869 | -0.170 | 0.659 | 0.133 | -0.112 | 0.045 | 0.092 | .937^*^ | 0.749 | -0.170 | 0.456 | 0.266 | 0.723 | 1 |
| DPPH | 0.314 | -0.481 | 0.468 | 0.701 | 0.699 | 0.740 | 0.715 | **0.972^**^** | -0.481 | **0.898^*^** | -0.481 | 0.620 | 0.286 | 0.251 | 0.201 | 0.388 | 0.838 | 0.871 | -0.481 | 0.466 | 0.257 | 1 |  |
| TPC | -0.408 | -0.131 | -0.657 | -0.091 | 0.845 | -0.263 | 0.096 | 0.227 | -0.131 | -0.024 | -0.131 | 0.722 | **0.943^*^** | 0.794 | **0.953^*^** | -0.714 | 0.468 | 0.613 | -0.131 | 0.863 | 1 |  |  |
| 20^1)^ | -0.559 | 0.151 | -0.468 | -0.088 | 0.772 | -0.126 | 0.485 | 0.378 | 0.151 | 0.240 | 0.151 | 0.554 | 0.700 | 0.521 | 0.855 | -0.623 | 0.697 | 0.834 | 0.151 | 1 |  |  |  |
| 19 | -0.813 | **1.000^**^** | -0.389 | -0.682 | -0.506 | -0.531 | 0.146 | -0.562 | **1.000^**^** | -0.348 | **1.000^**^** | -0.634 | -0.410 | -0.524 | -0.045 | -0.503 | -0.154 | -0.179 | 1 |  |  |  |  |
| 18 | -0.153 | -0.179 | 0.015 | 0.411 | 0.836 | 0.419 | 0.760 | 0.817 | -0.179 | 0.718 | -0.179 | 0.687 | 0.522 | 0.379 | 0.556 | -0.106 | **0.932^*^** | 1 |  |  |  |  |  |
| 17 | -0.187 | -0.154 | 0.007 | 0.601 | 0.728 | 0.607 | **0.893^*^** | 0.857 | -0.154 | 0.841 | -0.154 | 0.704 | 0.337 | 0.124 | 0.319 | -0.026 | 1 |  |  |  |  |  |  |
| 16 | 0.845 | -0.503 | **0.951^*^** | 0.620 | -0.242 | 0.728 | 0.121 | 0.424 | -0.503 | 0.513 | -0.503 | -0.135 | -0.526 | -0.345 | -0.713 | 1 |  |  |  |  |  |  |  |
| 15 | -0.384 | -0.045 | -0.578 | -0.300 | 0.758 | -0.430 | -0.023 | 0.106 | -0.045 | -0.158 | -0.045 | 0.535 | **0.915^*^** | 0.841 | 1 |  |  |  |  |  |  |  |  |
| 14 | 0.146 | -0.524 | -0.276 | -0.066 | 0.764 | -0.251 | -0.307 | 0.192 | -0.524 | -0.152 | -0.524 | 0.619 | **0.941^*^** | 1 |  |  |  |  |  |  |  |  |  |
| 13 | -0.101 | -0.410 | -0.489 | -0.002 | 0.871 | -0.206 | -0.101 | 0.262 | -0.410 | -0.053 | -0.410 | 0.767 | 1 |  |  |  |  |  |  |  |  |  |  |
| 12 | 0.091 | -0.634 | -0.219 | 0.612 | **0.920^*^** | 0.423 | 0.330 | 0.705 | -0.634 | 0.493 | -0.634 | 1 |  |  |  |  |  |  |  |  |  |  |  |
| 11 | -0.813 | **1.000^**^** | -0.389 | -0.682 | -0.506 | -0.531 | 0.146 | -0.562 | **1.000^**^** | -0.348 | 1 |  |  |  |  |  |  |  |  |  |  |  |  |
| 10 | 0.256 | -0.348 | 0.508 | 0.844 | 0.443 | **0.917^*^** | 0.858 | **0.939^*^** | -0.348 | 1 |  |  |  |  |  |  |  |  |  |  |  |  |  |
| 9 | -0.813 | **1.000^**^** | -0.389 | -0.682 | -0.506 | -0.531 | 0.146 | -0.562 | 1 |  |  |  |  |  |  |  |  |  |  |  |  |  |  |
| 8 | 0.346 | -0.562 | 0.431 | 0.839 | 0.692 | 0.842 | 0.722 | 1 |  |  |  |  |  |  |  |  |  |  |  |  |  |  |  |
| 7 | -0.261 | 0.146 | 0.171 | 0.520 | 0.349 | 0.637 | 1 |  |  |  |  |  |  |  |  |  |  |  |  |  |  |  |  |
| 6 | 0.510 | -0.531 | 0.626 | **0.960^**^** | 0.250 | 1 |  |  |  |  |  |  |  |  |  |  |  |  |  |  |  |  |  |
| 5 | 0.003 | -0.506 | -0.203 | 0.392 | 1 |  |  |  |  |  |  |  |  |  |  |  |  |  |  |  |  |  |  |
| 4 | 0.527 | -0.682 | 0.468 | 1 |  |  |  |  |  |  |  |  |  |  |  |  |  |  |  |  |  |  |  |
| 3 | 0.783 | -0.389 | 1 |  |  |  |  |  |  |  |  |  |  |  |  |  |  |  |  |  |  |  |  |
| 2 | -0.813 | 1 |  |  |  |  |  |  |  |  |  |  |  |  |  |  |  |  |  |  |  |  |  |
| 1 | 1 |  |  |  |  |  |  |  |  |  |  |  |  |  |  |  |  |  |  |  |  |  |  |

** means correlated between two variables (*p* < 0.01)

* means correlated between two variables (*p* < 0.05)

^1)^ Compound codes correspond to those listed in Table 1.

**Table S2(B).** Pearson’s correlations among individual anthocyanin compounds, total phenolic content (TPC), and antioxidant activities (ABTS and DPPH) of the inner layer.

| *Inner layer* | 1 | 2 | 3 | 4 | 5 | 6 | 7 | 8 | 9 | 10 | 11 | 12 | 13 | 14 | 15 | 16 | 17 | 18 | 19 | 20 | TPC | DPPH | ABTS |
| --- | --- | --- | --- | --- | --- | --- | --- | --- | --- | --- | --- | --- | --- | --- | --- | --- | --- | --- | --- | --- | --- | --- | --- |
| ABTS | **-0.976^**^** | 0.501 | **-0.899^*^** | -0.480 | 0.467 | -0.494 | 0.248 | 0.037 | 0.501 | -0.111 | 0.501 | 0.441 | 0.396 | 0.508 | 0.628 | **-0.912^*^** | 0.060 | 0.296 | 0.501 | 0.678 | 0.206 | 0.250 | 1 |
| DPPH | -0.069 | -0.596 | -0.224 | 0.650 | **0.906^*^** | 0.499 | 0.380 | **0.881^*^** | -0.596 | 0.661 | -0.596 | 0.783 | 0.638 | 0.462 | 0.582 | -0.131 | 0.819 | 0.850 | -0.596 | 0.707 | **0.977^**^** | 1 |  |
| TPC | -0.006 | -0.696 | -0.250 | 0.628 | **0.890^*^** | 0.391 | 0.183 | 0.803 | -0.696 | 0.519 | -0.696 | 0.866 | 0.760 | 0.590 | 0.653 | -0.162 | 0.707 | 0.755 | -0.696 | 0.707 | 1 |  |  |
| 20^1)^ | -0.569 | -0.082 | -0.529 | -0.061 | **0.932^*^** | -0.085 | 0.305 | 0.605 | -0.082 | 0.292 | -0.082 | 0.868 | 0.794 | 0.790 | **0.940^*^** | -0.523 | 0.540 | 0.758 | -0.082 | 1 |  |  |  |
| 19 | -0.676 | **1.000^**^** | -0.275 | -0.814 | -0.352 | -0.484 | 0.280 | -0.478 | **1.000^**^** | -0.281 | **1.000^**^** | -0.488 | -0.476 | -0.271 | -0.159 | -0.361 | -0.349 | -0.248 | 1 |  |  |  |  |
| 18 | -0.204 | -0.248 | -0.039 | 0.423 | **0.898^*^** | 0.569 | 0.736 | **0.957^*^** | -0.248 | 0.845 | -0.248 | 0.580 | 0.401 | 0.277 | 0.525 | 0.010 | **0.956^*^** | 1 |  |  |  |  |  |
| 17 | 0.025 | -0.349 | 0.181 | 0.619 | 0.765 | 0.783 | 0.777 | **0.983^**^** | -0.349 | **0.959^*^** | -0.349 | 0.402 | 0.207 | 0.038 | 0.277 | 0.248 | 1 |  |  |  |  |  |  |
| 16 | 0.860 | -0.361 | **0.991^**^** | 0.499 | -0.294 | 0.708 | 0.152 | 0.226 | -0.361 | 0.437 | -0.361 | -0.467 | -0.494 | -0.615 | -0.604 | 1 |  |  |  |  |  |  |  |
| 15 | -0.505 | -0.159 | -0.596 | -0.168 | 0.822 | -0.321 | -0.035 | 0.388 | -0.159 | -0.004 | -0.159 | **0.927^*^** | **0.925^*^** | **0.949^*^** | 1 |  |  |  |  |  |  |  |  |
| 14 | -0.371 | -0.271 | -0.607 | -0.179 | 0.661 | -0.463 | -0.344 | 0.182 | -0.271 | -0.247 | -0.271 | **0.913^*^** | **0.969^**^** | 1 |  |  |  |  |  |  |  |  |  |
| 13 | -0.225 | -0.476 | -0.514 | 0.069 | 0.756 | -0.250 | -0.283 | 0.360 | -0.476 | -0.071 | -0.476 | **0.975^**^** | 1 |  |  |  |  |  |  |  |  |  |  |
| 12 | -0.261 | -0.488 | -0.504 | 0.188 | 0.875 | -0.085 | -0.081 | 0.535 | -0.488 | 0.132 | -0.488 | 1 |  |  |  |  |  |  |  |  |  |  |  |
| 11 | -0.676 | 1.000^**^ | -0.275 | -0.814 | -0.352 | -0.484 | 0.280 | -0.478 | **1.000^**^** | -0.281 | 1 |  |  |  |  |  |  |  |  |  |  |  |  |
| 10 | 0.156 | -0.281 | 0.370 | 0.662 | 0.551 | .900^*^ | 0.838 | **0.904^*^** | -0.281 | 1 |  |  |  |  |  |  |  |  |  |  |  |  |  |
| 9 | -0.676 | **1.000^**^** | -0.275 | -0.814 | -0.352 | -0.484 | 0.280 | -0.478 | 1 |  |  |  |  |  |  |  |  |  |  |  |  |  |  |
| 8 | 0.076 | -0.478 | 0.157 | 0.649 | 0.831 | 0.741 | 0.653 | 1 |  |  |  |  |  |  |  |  |  |  |  |  |  |  |  |
| 7 | -0.290 | 0.280 | 0.128 | 0.205 | 0.406 | 0.601 | 1 |  |  |  |  |  |  |  |  |  |  |  |  |  |  |  |  |
| 6 | 0.537 | -0.484 | 0.626 | 0.847 | 0.251 | 1 |  |  |  |  |  |  |  |  |  |  |  |  |  |  |  |  |  |
| 5 | -0.321 | -0.352 | -0.339 | 0.302 | 1 |  |  |  |  |  |  |  |  |  |  |  |  |  |  |  |  |  |  |
| 4 | 0.615 | -0.814 | 0.383 | 1 |  |  |  |  |  |  |  |  |  |  |  |  |  |  |  |  |  |  |  |
| 3 | 0.827 | -0.275 | 1 |  |  |  |  |  |  |  |  |  |  |  |  |  |  |  |  |  |  |  |  |
| 2 | -0.676 | 1 |  |  |  |  |  |  |  |  |  |  |  |  |  |  |  |  |  |  |  |  |  |
| 1 | 1 |  |  |  |  |  |  |  |  |  |  |  |  |  |  |  |  |  |  |  |  |  |  |

** means correlated between two variables (*p* < 0.01)

* means correlated between two variables (*p* < 0.05)

^1)^ Compound codes correspond to those listed in Table 1.

**Table S2(C).** Pearson’s correlations among individual non-anthocyanin phenolic compounds, total phenolic content (TPC), and antioxidant activities (ABTS and DPPH) of the outer layer.

| *Outer layer* | g | f | h | m | b | o | a | c | n | p | r | e | i | d | j | k | l | q | TPC | DPPH | ABTS |
| --- | --- | --- | --- | --- | --- | --- | --- | --- | --- | --- | --- | --- | --- | --- | --- | --- | --- | --- | --- | --- | --- |
| ABTS | -0.330 | -0.384 | 0.111 | -0.256 | -0.181 | -0.332 | -0.379 | 0.274 | 0.446 | **-0.897^*^** | -0.685 | -0.116 | -0.111 | 0.668 | 0.578 | **0.892^*^** | -0.338 | 0.469 | 0.279 | 0.781 | 1 |
| DPPH | -0.438 | -0.844 | 0.242 | -0.657 | 0.085 | -0.725 | -0.350 | 0.442 | -0.012 | -0.792 | **-0.963^**^** | 0.352 | 0.387 | 0.682 | 0.759 | 0.877 | 0.243 | 0.465 | 0.255 | 1 |  |
| TPC | 0.112 | 0.011 | **0.947^*^** | -0.457 | -0.508 | -0.034 | -0.645 | 0.529 | 0.182 | 0.041 | -0.103 | 0.637 | 0.595 | 0.864 | 0.805 | 0.597 | -0.489 | **0.961^**^** | 1 |  |  |
| q^1)^ | -0.133 | -0.128 | 0.866 | -0.463 | -0.341 | -0.096 | -0.778 | 0.456 | 0.098 | -0.217 | -0.282 | 0.653 | 0.618 | **0.961^**^** | **0.915^*^** | 0.765 | -0.408 | 1 |  |  |  |
| l | -0.336 | -0.627 | -0.296 | -0.277 | 0.680 | -0.472 | 0.226 | -0.099 | -0.745 | 0.005 | -0.359 | 0.340 | 0.398 | -0.325 | -0.079 | -0.222 | 1 |  |  |  |  |
| k | -0.331 | -0.524 | 0.488 | -0.543 | -0.200 | -0.453 | -0.584 | 0.476 | 0.238 | -0.745 | -0.755 | 0.336 | 0.338 | **0.912^*^** | **0.884^*^** | 1 |  |  |  |  |  |
| j | -0.287 | -0.507 | 0.765 | -0.675 | -0.157 | -0.423 | -0.705 | 0.536 | -0.044 | -0.423 | -0.621 | 0.728 | 0.722 | **0.967^**^** | 1 |  |  |  |  |  |  |
| d | -0.225 | -0.331 | 0.767 | -0.551 | -0.294 | -0.280 | -0.736 | 0.508 | 0.145 | -0.449 | -0.518 | 0.581 | 0.561 | 1 |  |  |  |  |  |  |  |
| i | -0.229 | -0.456 | 0.703 | -0.630 | 0.156 | -0.341 | -0.519 | 0.353 | -0.574 | 0.119 | -0.316 | **0.997^**^** | 1 |  |  |  |  |  |  |  |  |
| e | -0.224 | -0.395 | 0.729 | -0.596 | 0.135 | -0.282 | -0.558 | 0.335 | -0.560 | 0.139 | -0.266 | 1 |  |  |  |  |  |  |  |  |  |
| r | 0.272 | **0.935^*^** | -0.162 | 0.738 | -0.030 | 0.872 | 0.090 | -0.539 | -0.041 | 0.700 | 1 |  |  |  |  |  |  |  |  |  |  |
| p | 0.649 | 0.460 | 0.214 | 0.082 | -0.258 | 0.285 | 0.380 | 0.052 | -0.098 | 1 |  |  |  |  |  |  |  |  |  |  |  |
| n | 0.631 | 0.177 | 0.134 | -0.081 | -0.857 | -0.094 | 0.326 | 0.476 | 1 |  |  |  |  |  |  |  |  |  |  |  |  |
| c | 0.575 | -0.535 | 0.700 | **-0.898^*^** | -0.730 | -0.751 | 0.178 | 1 |  |  |  |  |  |  |  |  |  |  |  |  |  |
| a | 0.659 | -0.062 | -0.439 | -0.024 | -0.246 | -0.286 | 1 |  |  |  |  |  |  |  |  |  |  |  |  |  |  |
| o | -0.144 | **0.938^*^** | -0.236 | **0.888^*^** | 0.171 | 1 |  |  |  |  |  |  |  |  |  |  |  |  |  |  |  |
| b | -0.825 | -0.143 | -0.555 | 0.359 | 1 |  |  |  |  |  |  |  |  |  |  |  |  |  |  |  |  |
| m | -0.240 | 0.802 | -0.652 | 1 |  |  |  |  |  |  |  |  |  |  |  |  |  |  |  |  |  |
| h | 0.285 | -0.143 | 1 |  |  |  |  |  |  |  |  |  |  |  |  |  |  |  |  |  |  |
| e | 0.202 | 1 |  |  |  |  |  |  |  |  |  |  |  |  |  |  |  |  |  |  |  |
| g | 1 |  |  |  |  |  |  |  |  |  |  |  |  |  |  |  |  |  |  |  |  |

** means correlated between two variables (*p* < 0.01)

* means correlated between two variables (*p* < 0.05)

^1)^ Compound codes correspond to those listed in Table 3.

**Table S2(D)** Pearson’s correlations among individual non-anthocyanin phenolic compounds, total phenolic content (TPC), and antioxidant activities (ABTS and DPPH) of the inner layer.

| *Inner layer* | g | f | h | m | b | o | a | c | n | p | r | e | i | d | j | l | q | TPC | DPPH | ABTS |
| --- | --- | --- | --- | --- | --- | --- | --- | --- | --- | --- | --- | --- | --- | --- | --- | --- | --- | --- | --- | --- |
| ABTS | -0.123 | 0.117 | 0.469 | -0.366 | -0.189 | 0.339 | 0.053 | 0.483 | 0.084 | 0.315 | 0.431 | 0.149 | 0.634 | 0.672 | 0.815 | **-0.941^*^** | 0.829 | 0.753 | 0.728 | 1 |
| DPPH | -0.582 | -0.468 | 0.374 | -0.832 | 0.024 | 0.236 | 0.219 | 0.517 | -0.616 | 0.139 | 0.173 | 0.576 | 0.630 | **0.940^*^** | **0.901^*^** | -0.501 | **0.974^**^** | **0.979^**^** | 1 |  |
| TPC | -0.665 | -0.412 | 0.248 | -0.878 | 0.186 | 0.369 | 0.025 | 0.370 | -0.566 | 0.336 | 0.321 | 0.649 | 0.772 | **0.890^*^** | **0.943^*^** | -0.531 | **0.936^*^** | 1 |  |  |
| q^1)^ | -0.393 | -0.331 | 0.527 | -0.687 | -0.168 | 0.167 | 0.325 | 0.647 | -0.459 | 0.059 | 0.167 | 0.420 | 0.557 | **0.941^*^** | **0.889^*^** | -0.639 | 1 |  |  |  |
| l | -0.050 | -0.207 | -0.356 | 0.100 | 0.246 | -0.430 | 0.025 | -0.352 | -0.372 | -0.257 | -0.307 | 0.160 | -0.467 | -0.404 | -0.579 | 1 |  |  |  |  |
| j | -0.496 | -0.100 | 0.383 | -0.777 | 0.118 | 0.232 | 0.008 | 0.425 | -0.423 | 0.459 | 0.579 | 0.686 | 0.840 | **0.889^*^** | 1 |  |  |  |  |  |
| d | -0.372 | -0.280 | 0.618 | -0.725 | -0.180 | -0.091 | 0.430 | 0.714 | -0.626 | 0.022 | 0.238 | 0.598 | 0.519 | 1 |  |  |  |  |  |  |
| i | -0.663 | -0.019 | -0.117 | -0.746 | 0.577 | 0.570 | -0.533 | -0.122 | -0.241 | 0.856 | 0.770 | 0.726 | 1 |  |  |  |  |  |  |  |
| e | -0.728 | -0.252 | -0.045 | -0.862 | 0.556 | 0.082 | -0.215 | -0.013 | -0.746 | 0.537 | 0.554 | 1 |  |  |  |  |  |  |  |  |
| r | -0.138 | 0.583 | 0.067 | -0.274 | 0.341 | 0.140 | -0.473 | -0.097 | 0.126 | 0.840 | 1 |  |  |  |  |  |  |  |  |  |
| p | -0.488 | 0.249 | -0.437 | -0.411 | 0.747 | 0.607 | -0.847 | -0.529 | 0.094 | 1 |  |  |  |  |  |  |  |  |  |  |
| n | 0.688 | 0.724 | -0.048 | 0.804 | -0.263 | 0.108 | -0.247 | -0.214 | 1 |  |  |  |  |  |  |  |  |  |  |  |
| c | 0.344 | 0.058 | **0.967^**^** | -0.056 | -0.813 | -0.580 | **0.884^*^** | 1 |  |  |  |  |  |  |  |  |  |  |  |  |
| a | 0.457 | -0.085 | 0.824 | 0.161 | -0.865 | -0.754 | 1 |  |  |  |  |  |  |  |  |  |  |  |  |  |
| o | -0.624 | -0.332 | -0.659 | -0.377 | 0.669 | 1 |  |  |  |  |  |  |  |  |  |  |  |  |  |  |
| b | -0.786 | -0.305 | -0.840 | -0.523 | 1 |  |  |  |  |  |  |  |  |  |  |  |  |  |  |  |
| m | **0.903^*^** | 0.601 | 0.084 | 1 |  |  |  |  |  |  |  |  |  |  |  |  |  |  |  |  |
| h | 0.497 | 0.307 | 1 |  |  |  |  |  |  |  |  |  |  |  |  |  |  |  |  |  |
| e | 0.708 | 1 |  |  |  |  |  |  |  |  |  |  |  |  |  |  |  |  |  |  |
| g | 1 |  |  |  |  |  |  |  |  |  |  |  |  |  |  |  |  |  |  |  |

** means correlated between two variables (*p* < 0.01)

* means correlated between two variables (*p* < 0.05)

^1)^ Compound codes correspond to those listed in Table 3.
